# The MIR181A2HG/miR‐5680/VCAN‐CD44 Axis Regulates Gastric Cancer Lymph Node Metastasis by Promoting M2 Macrophage Polarization

**DOI:** 10.1002/cam4.70600

**Published:** 2025-01-16

**Authors:** Weijie Zang, Yongpu Yang, Junjie Chen, Qinsheng Mao, Wanjiang Xue, Yilin Hu

**Affiliations:** ^1^ Department of Gastrointestinal Surgery Affiliated Hospital and Medical School of Nantong University Nantong China; ^2^ Research Center of Clinical Medicine Affiliated Hospital of Nantong University Nantong China; ^3^ Nantong Key Laboratory of Gastrointestinal Oncology Nantong China; ^4^ Department of General Surgery The First Affiliated Hospital, Army Medical University Chongqing China; ^5^ Department of Graduate School Dalian Medical University Dalian China

**Keywords:** CD44, gastric cancer, lymphatic metastasis, macrophage, MIR181A2HG, miR‐5680, VCAN

## Abstract

**Background:**

Lymphatic metastasis in gastric cancer (GC) profoundly influences its prognosis, but the precise mechanism remains elusive. In this study, we identified the long noncoding RNA MIR181A2HG as being upregulated in GC and associated with LNs metastasis and prognosis.

**Methods:**

The expression of MIR181A2HG in GC was identified through bioinformatics screening analysis and qRT‐PCR validation. Both in vitro and in vivo functional studies revealed that MIR181A2HG facilitates lymphangiogenesis and lymphatic metastasis. Techniques such as immunofluorescence, immunohistochemistry, qRT‐PCR, ELISA, CHIP, RNA‐pulldown, luciferase reporter assay, and Co‐IP were employed to investigate the mechanism of MIR181A2HG in LNs metastasis of GC.

**Results:**

MIR181A2HG overexpressed in GC signifies an unfavorable prognosis and drives M2 polarization of TAMs enhancing lymphangiogenesis. Mechanistically, MIR181A2HG/miR‐5680 axis as a novel ceRNA regulatory axis to upregulate versican (VCAN). On one hand, VCAN interacts with CD44 receptors on the surface of TAMs through paracrine secretion, promoting M2 macrophage polarization and subsequently enhancing the secretion of VEGF‐C, ultimately facilitating lymphangiogenesis. On the other hand, VCAN binds to CD44 receptors on the surface of GC cells through autocrine secretion, activating the Hippo pathway and upregulating SP1, thereby promoting the transcription of MIR181A2HG and establishing a feedback loop driving lymphatic metastasis.

**Conclusion:**

This study highlights the pivotal role of MIR181A2HG in GC progression and LNs metastasis. MIR181A2HG‐based targeted therapy would represent a novel strategy for GC.

**Abbreviations**
ceRNAs: competing endogenous RNAs
ChIP: chromatin immunoprecipitation
Co‐IP: co‐immunoprecipitation
DFI: disease‐free interval
DSS: disease‐specific survival
GC: gastric cancer
GEO: gene expression omnibus
HE: hematoxylin and eosin
HLECs: human lymphatic endothelial cells
IHC: immunohistochemistry
lncRNAs: long con‐coding RNAs
LNs: lymph nodes
MLVD: microlymphatic vessel density
OS: overall survival
PFI: progression‐free interval
PMA: Phorbol‐12‐myristate‐13‐acetate
qRT‐PCR: quantitative real‐time PCR
SP1: specificity protein 1
SRF: serum response factor
ssGSEA: single‐sample gene set enrichment analysis
TAMs: tumor‐associated macrophages
TBP: TATA‐box binding protein
TME: tumor microenvironment
VCAN: versican
VEGF‐C: vascular endothelial growth factor C

## 1 Introduction

According to global cancer statistics, gastric cancer (GC) has an incidence rate of 5.7% and a mortality rate of 8.2%. This ranks GC as the fifth most common cause of cancer‐related deaths globally [1]. GC patients commonly experience a poor prognosis primarily due to tumor metastasis and recurrence [2]. Among various factors influencing GC progression, tumor‐associated macrophages (TAMs) play a crucial role [3]. As a consequence of lymphangiogenesis, high lymphatic vessel density has been reported to be associated with an unfavorable prognosis in GC [4]. Therefore, investigating the mechanisms of lymphangiogenesis in GC is of great significance for exploring new therapeutic targets and inhibiting lymphatic dissemination.

One of the most well‐known lymphangiogenic factors involved in the process of tumor‐associated lymphangiogenesis is vascular endothelial growth factor C (VEGF‐C) [5]. VEGF‐C plays a crucial role in promoting the growth and remodeling of lymphatic vessels within the tumor microenvironment (TME) [6]. TAMs have been identified as major sources of VEGF‐C production within the TME [7]. While several studies have investigated the mechanisms underlying tumor‐associated lymphangiogenesis, the majority of them have focused on cancer‐intrinsic pathways that regulate the secretion of VEGF‐C [8, 9, 10]. However, the precise involvement and regulatory functions of TAMs in secreting VEGF‐C, which are employed by GC cells, in the context of tumor‐associated lymphangiogenesis, remain unclear.

Long non‐coding RNAs (lncRNAs) have garnered significant attention due to their crucial roles in tumor biology [11, 12, 13, 14]. These RNA molecules, exceeding 200 nucleotides in length, play important functions in the metastasis of tumor cells by participating in lymphangiogenesis [11, 15]. For instance, LNMAT2 has been found to promote the migration and lymphangiogenesis of human lymphatic endothelial cells through the regulation of the VEGF signaling pathway [16]. Additionally, the upregulation of LINC00665 has been observed in various cancers and is associated with lymphangiogenesis and poor prognosis [17]. However, the precise mechanism by which lncRNAs contribute to lymphangiogenesis in GC remains unexplored and necessitates further investigation.

Here, we identified a differentially expressed MIR181A2HG using online databases. Subsequently, we evaluated its expression levels in GC and examined its correlation with lymphangiogenesis. Furthermore, we have observed that MIR181A2HG promotes lymphangiogenesis by inducing M2 polarization of macrophages. This leads to the accelerated secretion of VEGF‐C in an autocrine and paracrine manner, which is dependent on the expression of VCAN. Overall, our findings demonstrate that MIR181A2HG‐mediated VCAN promotes M2 polarization of macrophages, thereby facilitating lymphangiogenesis in the tumor microenvironment of GC.

## 2 Materials and Methods

### 2.1 Clinical Samples

Between 2010 and 2011, a total of 108 gastric cancer (GC) tissue samples, along with their corresponding adjacent normal tissues, were collected by the Department of General Surgery at the Affiliated Hospital of Nantong University in Nantong, China. Table 1 presents the comprehensive clinical information of the GC patients. Patients were monitored until August 2015, with an average observation time of 56.52 months (spanning 1.81–66.67 months). Every GC diagnosis was confirmed by two independent pathologists, and none of the patients underwent radiotherapy, chemotherapy, or immunotherapy before their primary surgery.

**TABLE 1:** Correlation between MIR181A2HG expression in GC tissues and clinicopathological features of GC patients.

Clinicopathological parameter	*N*	MIR181A2HG expression		*p*
		Low (54)	High (54)	
Gender				
Male	74	39 (52.7%)	35 (47.3%)	0.407
Female	34	15 (44.1%)	19 (55.9%)	
Age (years)				
< 60	48	23 (47.9%)	25 (52.1%)	0.699
≥ 60	60	31 (51.7%)	29 (48.3%)	
Degree of differentiation				
Well	6	3 (50.0%)	3 (50.0%)	1.000
Moderate/poor	102	51 (50.0%)	51 (50.0%)	
Tumor diameter (cm)				
< 5	72	42 (58.3%)	30 (41.7%)	0.014
≥ 5	36	12 (33.3%)	24 (66.7%)	
TNM stage				
I + II	71	45 (63.4%)	26 (36.6%)	< 0.001
III	37	9 (24.3%)	28 (75.5%)	
Depth of invasion				
T1 + T2	56	31 (55.4%)	25 (44.6%)	0.248
T3 + T4	52	23 (44.2%)	29 (55.8%)	
Lymph node metastasis				
Negative	59	42 (71.2%)	17 (28.8%)	< 0.001
Positive	49	12 (24.5%)	37 (75.5%)	
Lymphatic microvessel density (MLVD)				
High	58	20 (34.5%)	38 (65.5%)	0.001
Low	50	34 (68.0%)	16 (32.0%)	

*Note: p* value < 0.05 was considered statistically significant.

### 2.2 Cell Culture and Associated Biochemicals

The gastric mucosa cell line GES‐1, seven gastric cancer cell lines (MKN‐45, BGC‐823, SGC‐7901, AGS, and HGC‐27), THP‐1 and Human Lymphatic Endothelial Cells (HLECs) purchased from ScienCell Research Laboratories (California, USA). The GES‐1 and GC cell lines are cultured in RPMI‐1640 medium from ScienCell Research Laboratories, supplemented with 10% fetal bovine serum (Clark, Shanghai, China) and 100 U/mL penicillin–streptomycin (Life Technologies, Shanghai, China). On the other hand, HLECs are cultured in Endothelial Cell Medium (ECM) supplemented with 10% serum and 0.1% endothelial cell growth factor. recombinant VEGF‐C and VCAN protein were purchased from MedChemExpress (New Jersey, USA), Calcein AM were purchased from Beyotime (Shanghai, China), and Phorbol‐12‐myristate‐13‐acetate (PMA) was sourced from SIGMA (Missouri, USA).

### 2.3 Plasmids, siRNAs and Cell Transfections

Full‐length cDNAs of human VCAN (versican) and SP1 (specificity protein 1) were cloned into GV141 vector (GeneChem). The wild type MIR181A2HG promoter (WT) and a promoter with mutated SP1‐binding sites (Mut) were cloned into GV248 luciferase plasmid vector (GeneChem). siRNA sequences corresponding to si‐MIR181A2HG#1, si‐MIR181A2HG#2, si‐MIR181A2HG#3, si‐VCAN, were synthesized by Genepharma (Suzhou, China). The relevant sequences can be found in Table S1. has‐miR‐5680 mimic, inhibitor and negative control (NC) was provided by Genepharma. GC cells were introduced with the plasmids and previously mentioned oligonucleotides using jetPRIME (Polyplus, Strasbourg, France) as per the guidelines provided by the manufacturer. Neomycin (G418; Roche, Indianapolis, IN, USA) was used to screen stably transfected GC cells for more than 2 weeks.

### 2.4 RNA Isolation and Quantitative Real‐Time PCR (qRT‐PCR)

RNA isolation and qRT‐PCR were performed previously. Table S2 details the sequences of the primers [18]. For normalization purposes, GAPDH and U6 mRNA served as internal references. Sangon Biotech (Shanghai, China) was responsible for the synthesis of all primers.

### 2.5 Western Blot

Protein extraction and western blotting were performed as described in our previous publication [19]. The following primary antibodies were used in our western blotting analysis: CD44(15675‐1‐AP), anti‐GAPDH(60004‐1‐Ig), anti‐YAP1 (66900‐1‐Ig), anti‐p‐YAP1 (29018‐1‐AP), SP1 (21962‐1‐AP) and anti‐IgG (A21020‐1) from Proteintech (Wuhan, China); Anti‐CD163 (ab182422) and anti‐CD68 (ab283654) from Abcam (Cambridge Science Park, England); VCAN (DF10007), TEAD1 (DF3141) and VEGF‐C (DF7011) from Affinity (Changzhou, China).

### 2.6 Tube Formation and Transwell Assays

After a 24‐h treatment with conditioned medium (CM) from both the treatment and control groups, HLECs were seeded into a 96‐well plate (NEST, Wuxi, China). Two hours post‐seeding, tube‐like structures were observed. Subsequently, the HLECs were fluorescently stained with Calcein AM (Beyotime, Shanghai, China). The tube formation capability was quantitatively assessed using ImageJ and the Angiogenesis Analyzer plugin. The transwell assay was conducted as described in the previous literature [20].

### 2.7 Immunofluorescence

HLECs seeded onto cell climbing tablets in a 24‐well plate underwent fixation, permeabilization, and blocking, and were incubated overnight at 4°C with primary antibodies targeting specific proteins, the same as those used in the Western Blot. Subsequently, the cells were incubated with Alexa Fluor 594‐conjugated goat anti‐rabbit IgG (ABclonal, Wuhan, China) and Alexa Fluor 488‐conjugated goat anti‐mouse IgG (ABclonal, Wuhan, China). They were then counterstained with 4′,6‐diamidino‐2‐phenylindole (DAPI, Invitrogen, California, USA). Tissue immunofluorescence was performed as above [21].

### 2.8 Luciferase Reporter Assay

Appropriate plasmids were transfected into SGC‐7901 and MKN‐45 cells in 24‐well plates for 48 h. Luciferase reporter gene plasmids were ordered from GeneChem. luciferase activity was measured with a dual‐luciferase assay kit (Beyotime, shanghai, China). Renilla luciferase was used as an internal control to normalize luciferase activity.

### 2.9 Enzyme‐Linked Immunosorbent Assay (ELISA) Assay

After the designated treatment, macrophages were starved for 24 h, and the CM was collected and centrifuged at 1500 rpm for 5 min at 4°C. Whole blood was centrifuged at 1000 *g* for 10 min at 4°C, and plasma was then collected. The expression level of VEGF‐C and VCAN in the supernatant was determined using an ELISA kit, following the manufacturer's instructions.

### 2.10 Hematoxylin and Eosin (HE) Staining and Immunohistochemistry (IHC) Assays

The methods for immunohistochemistry and H&E staining were as previously described [22, 23]. Criteria for lymphatic vessel identification: positive staining for both D2‐40 and LYVE‐1 is localized to the membrane and cytoplasm of lymphatic endothelial cells, manifesting as brown‐yellow granules. Counting method: D2‐40 and LYVE‐1 are specifically expressed in lymphatic vessels. Based on the method described by Dong et al., MLVD in the tissue was assessed [24]. Initially, regions rich in lymphatic vessels were chosen under 40× magnification. Subsequently, the number of microlymphatic vessels was counted under a 400× magnification. For each sample, three fields were counted, with the average of these counts determining the microlymphatic vessel density for that specific case.

### 2.11 Subcellular Fractionation

We utilized the Subcellular Protein Extraction Kit (Sangon Biotech) and another kit from Norgen Biotek, Canada, designed for cytoplasmic and nuclear RNA isolation, executing the process in line with the manufacturer's specifications. U6 served as the nuclear reference, while GAPDH acted as the cytoplasmic reference.

### 2.12 Chromatin Immunoprecipitation (ChIP) Assays

The ChIP assay was carried out as previously described [18]. Anti‐SP1 (21962‐1‐AP, Proteintech) and a control IgG antibody (MultiSciences, Hangzhou, China) were used for immunoprecipitation. PCR analysis of the ChIP‐obtained DNA was performed to determine the binding of SP1 to the MIR181A2HG promoter, normalized against the control IgG. Primer details are provided in Table S2.

### 2.13 Co‐Immunoprecipitation (Co‐IP) Analysis

Cells were lysed to obtain the supernatant, which was then incubated overnight with either the control IgG or specific antibodies. Subsequently, Protein A + G agarose beads (Bioworld Technology, Louis Park, USA) were introduced and the mixture was incubated at 4°C for 2 h. After washing the resultant protein‐antibody complexes in PBS, the beads were centrifuged to clean them. The supernatant was then discarded, and the retained samples were analyzed using sodium dodecyl sulfate‐polyacrylamide gel electrophoresis.

### 2.14 RNA Pulldown

Template DNA of MIR181A2HG and miR‐5680 were subjected to in vitro transcription using Biotin RNA Labeling Mix in conjunction with T7 RNA polymerase (Roche, Switzerland). The synthesized RNA was subsequently purified utilizing the RNeasy Mini Kit (Qiagen, USA) as per the provided protocol. Beads bound with RNA were then incubated with SGC‐7901 or MKN‐45 cell lysates, followed by RNA elution, purification, and analysis via qRT‐PCR.

### 2.15 Popliteal Lymphatic Metastasis Model

Four‐week‐old male athymic nude mice were purchased from the Animal Center of the Medical College of Nantong University and were maintained under controlled temperature and humidity conditions. Experimental procedures were performed as described previously [25]. A total of 15 mice, spread across three groups, had their footpads inoculated with 100 μL of 1640 medium suspensions of GC cells that were transduced with si‐MIR181A2HG#1, si‐MIR181A2HG#2 or si‐NC. Four weeks post‐injection, lymphatic metastasis was monitored and imaged using a bioluminescence imaging system (PerkinElmer, IVIS Spectrum Imaging System). When tumors in the control set matched the dimensions of their counterparts in the test set, we assessed tumor progression and lymphatic dissemination. Primary tumors and popliteal LNs were extracted and embedded in paraffin. Serial sections of 4.0 mm were obtained and subsequently analyzed by IHC.

### 2.16 Statistical Analysis

Data were expressed as mean ± standard deviation (SD). All data were analyzed using SPSS 25.0 (Chicago, IL, USA). All experiments except animal experiments and IHC were performed at least three times. The overall survival rate (OS) and disease‐free survival rate (DFS) of GC patients were analyzed and calculated by the Kaplan–Meier and log‐rank methods [26]. Cox regression model was used to evaluate the prognostic factors associated with GC. The experimental groups were compared using the *t*‐test with *p* < 0.05 considered to indicate statistical significance.

### 2.17 Online Databases and Bioinformatics Analysis

Differential gene selection based on TCGA STAD (portal.gdc.cancer.gov) and GEO (GSE109476 and GSE54129, ncbi.nlm.nih.gov/geo). The TCGA STAD database is used to analyze the relationship between the expression level of MIR181A2HG and the prognosis of GC. All data are converted to Log_2_ (*n* + 1) values. The Subcellular Localization prediction database predicts (csbio.sjtu.edu.cn) the subcellular localization of MIR181A2HG. The Lncbase V2 Database predicts (diana.e‐ce.uth.gr) the downstream miR‐5680 and binding sites of MIR181A2HG. The online databases RNA22 (jefferson.edu), DIANA (diana.e‐ce.uth.gr), TargetMiner (hsls.pitt.edu), TargetScan (targetscan.org), and multiMIR (multimir.org) predict the downstream target genes and binding sites of miR‐5680. Genecard (genecards.org) selects the top 1000 macrophage‐related genes. Based on the ssGSEA algorithm provided in the R package‐GSVA (Hänzelmann et al., 2013) [27], the markers of macrophages provided in the Immunity article (Bindea et al.) [28] are used to calculate the immune infiltration of the corresponding cloud data. We performed immune infiltration analysis by calculating the correlation (Spearman) between VCAN mRNA (TPM) expression in the TCGA‐STAD dataset and the ssGSEA scores of the 24 immune cell types. The online databases GENEMANIA (genemania.org), STRING (string‐db.org), BIOGRID (thebiogrid.org), HINT (hint.yulab.org), and Hitpredict (hitpredict.org) predict the interacting proteins of VCAN. Based on online databases HTFtarget (bioinfo.life.hust.edu.cn/hTFtarget), HumanTFDB (bioinfo.life.hust.edu.cn/HumanTFDB), GTRD (gtrd20‐06.biouml.org), JASPAR (jaspar.genereg.net), TRANSFAC (gene‐regulation.com/pub/databases), and PAZER (pazar.info), the transcription factors of MIR181A2HG are predicted, and a correlation analysis is conducted based on the TCGA STAD database, followed by differential expression screening. The JASPAR database predicts the binding sites of SP1 and the promoter region of MIR181A2HG.

## 3 Results

### 3.1 MIR181A2HG Is Intricately Involved in GC Progression, Particularly LNs Metastasis

**FIGURE 1:**
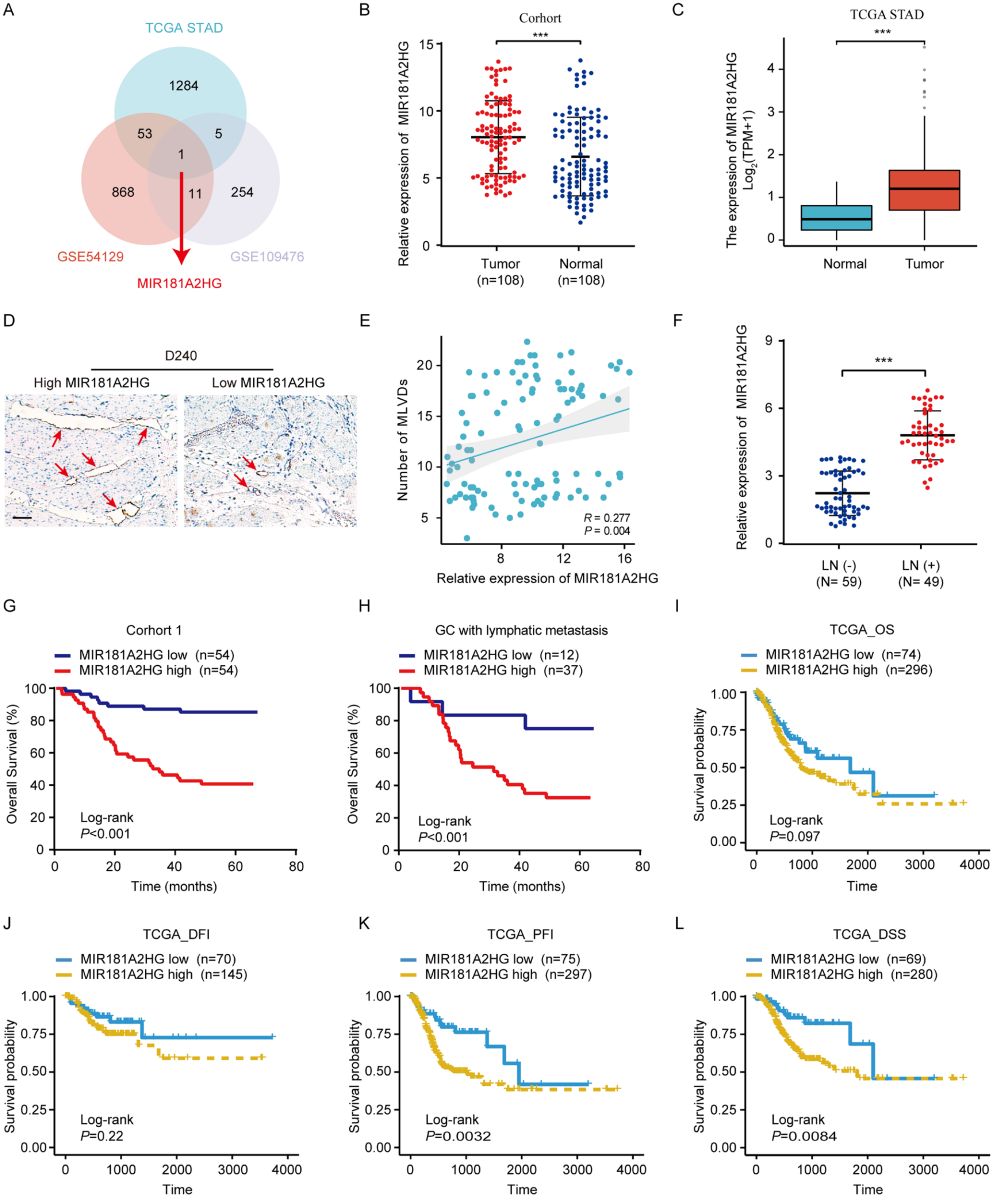
High expression of MIR181A2HG indicates poor prognosis of GC. (A) Venn diagram showing MIR181A2HG was found to be differentially expressed in the two databases (GSE109476 and GSE54129) and TCGA STAD database. (B) qRT‐PCR was used to detect the expression of MIR181A2HG in 108 matched GC samples. (C) The expression of MIR181A2HG between GC and normal tissues in TCGA STAD database. (D) Typical immunohistochemical images of D2‐40 (lymphatic marker) in different MIR181A2HG expression groups. (E) Correlation between the expression level of MIR181A2HG and MLVD (micro‐lymphatic vessel density) in 108 GC tissues (scale bar, 100 μm). (F) qRT‐PCR was used to detect the expression of MIR181A2HG in LNs metastasis positive group (LN^+^) and LNs metastasis negative group (LN^−^). (G, H) OS of GC patients (G) or GC patients with LNs metastasis (H) related to MIR181A2HG expression by Kaplan–Meier survival curve analysis (*p* < 0.001). (I–L) OS (I), DFI (J), PFI (K), or DSS (L) of GC patients related to MIR181A2HG expression by Kaplan–Meier survival curve analysis. Statistical significance was assessed by χ^2^ test. ***p < 0.001.

Tn order to investigate the lncRNAs closely associated with lymphatic metastasis in GC we capitalized on GEO datasets (GSE54129 and GSE109476) and TCGA‐STAD (Figure S1). We conducted differential expression analysis on GC tissues, singling out lncRNAs with significant differences (*p* < 0.01). Of these, only MIR181A2HG was consistently featured across all three datasets (Figure 1A). Expression levels of MIR181A2HG in a cohort of 108 GC tissues and corresponding normal GC tissues were analyzed, revealing significantly higher MIR181A2HG expressions in GC tissues than in their paired normal gastric counterparts (Figure 1B). TCGA STAD data confirmed these results (Figure 1C).

We subsequently analyzed the association between MIR181A2HG expression and diverse clinical characteristics in a cohort of 108 GC patients (Table 1). A notable correlation was observed between MIR181A2HG levels and factors such as LNs metastasis (*p* < 0.001), TNM stage (*p* < 0.001), and lymphatic microvessel density (MLVD, *p* = 0.001). Comprehensive logistic regression analysis reinforced that MIR181A2HG levels significantly tie with LNs metastasis (*p* = 0.002, odds ratio = 2.424, 95% CI: 0.375–15.660) and MLVD (*p* = 0.005, odds ratio = 3.628, 95% CI: 1.477–8.911). The IHC data underscored a direct positively relationship between MIR181A2HG expression and MLVD in the 108 GC patients (Figure 1D,E). Additionally, the expression level of MIR181A2HG in LNs metastasis positive group was significantly higher than that in LNs metastasis negative group (Figure 1F). Crucially, elevated levels of MIR181A2HG correlate with reduced overall survival (OS) in GC patients, including those with lymphatic metastasis (*p* < 0.01) (Figure 1G,H). Kaplan–Meier survival curve analysis indicated that an increase in MIR181A2HG levels adversely affected OS in the TCGA dataset (Figure 1I–L). Notably, there's a marked distinction between disease‐specific survival (DSS) and progression‐free interval (PFI). Both univariate and multivariate Cox assessments underscored that MIR181A2HG expression stands as an autonomous predictor for OS (Table 2). To sum up, our findings highlight the significant influence of MIR181A2HG on the advancement and lymphatic spread of GC.

**TABLE 2:** Univariate and multivariable analyses of OS of patients with GC.

Variable	OS		
	Univariate analysis	Multivariable analysis	
	*p*	*p*	HR (95% CI)
Gender			
Male (*n* = 74) vs. female (*n* = 34)	0.678		
Age (years)			
≤ 60 (*n* = 48) vs. > 60 (*n* = 60)	0.352		
Differentiation			
Well (*n* = 6) vs. moderate/poor (*n* = 102)	0.302		
Tumor diameter (cm)			
< 5 (*n* = 72) vs. ≥ 5 (36)	0.475		
Depth of invasion			
T1 + T2 (*n* = 56) vs. T3 + T4 (*n* = 52)	0.432		
Lymph node metastasis			
Negative (*n* = 59) vs. positive (*n* = 49)	< 0.001	0.599	0.772 (0.294–2.027)
MIR181A2HG expression			
Low (*n* = 54) vs. high (*n* = 54)	< 0.001	0.01	0.297 (0.126–0.699)
Lymphatic microvessel density (MLVD)			
Low (*n* = 50) vs. high (*n* = 58)	< 0.001	0.04	0.458 (0.216–0.971)

*Note: p* value < 0.05 was considered statistically significant.

### 3.2 MIR181A2HG Fosters Lymphatic Metastasis In Vivo

**FIGURE 2:**
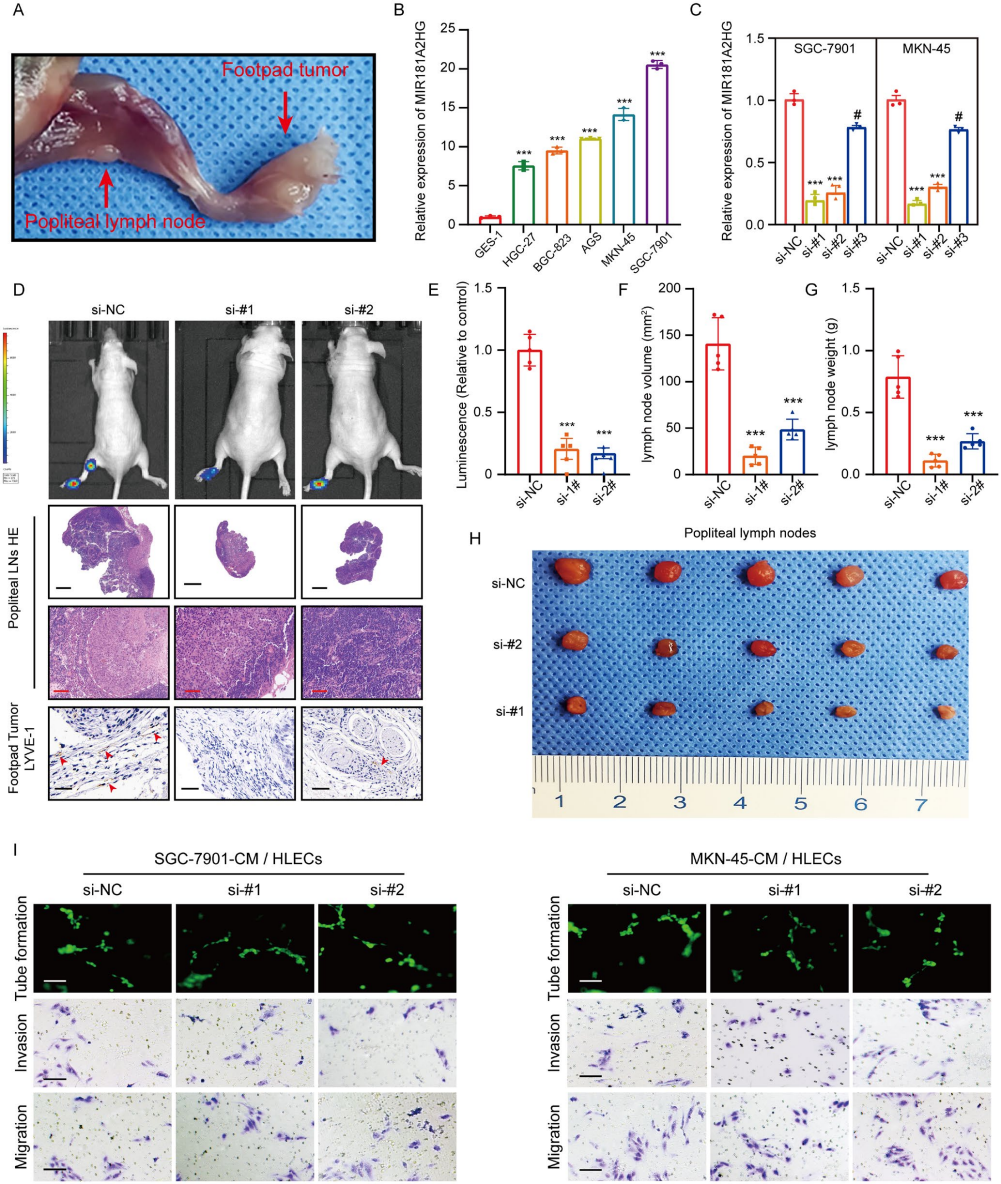
High expression of MIR181A2HG promotes lymphatic metastasis in vivo. (A) Representative image of a nude mouse popliteal LNs metastasis model. (B, C) qRT‐PCR detection of MIR181A2HG expression in different GC cells, normal gastric mucosal epithelial cells (B), and different treatment groups (C). (D) Representative images of cell bioluminescence, HE staining (popliteal LN, scale bar, 20 and 200 μm), and IHC of LYVE‐1, a marker of lymphatic vessels (footpad tumor, scale bar, 50 μm). (E) Quantification of the bioluminescence in popliteal LNs metastasis model. (F, G) Quantification of the volume (F) and weight (G) of metastatic popliteal LNs. (H) Representative images of metastatic popliteal LNs. (I) Representative images of tube formation and transwell migration of HLECs treated with conditioned medium from different treatment groups: Si‐NC (control group), si‐#1 (si‐MIR181A2HG‐1), si‐#2 (si‐MIR181A2HG‐2). Statistical significance was assessed using two‐tailed *t*‐tests. **p < 0.01, ***p < 0.001, ^#^ *p* > 0.05 (scale bar, 50 μm).

To delve deeper into MIR181A2HG's involvement in LNs metastasis in GC, we utilized a live‐model system in nude mice, replicating popliteal LN metastasis observed in GC, which emulates the directional metastasis of GC LNs (Figure 2A). Initially, qRT‐PCR was used to probe MIR181A2HG's expression level in different GC cells (Figure 2B). The SGC‐7901 and MKN‐45 cells, exhibiting the highest expression level, were selected for knockdown transfection: si‐#1, si‐#2, si‐#3 (Figure 2C). Upon reaching a footpad tumor size of 500 mm^3^, MIR181A2HG's impact on LNs metastasis was evaluated. MIR181A2HG knockdown group can significantly inhibit popliteal lymphatic metastasis of GC cells, as determined by fluorescence intensity and HE staining (Figure 2D,E). Meanwhile, the popliteal LNs volume and weight in MIR181A2HG knockdown group was significantly smaller than that in control group (Figure 2F–H).

Prior research has confirmed that lymphangiogenesis associated with tumors primarily influences tumor lymph node metastasis [15]. Concurrently, IHC data from the footpad tumor revealed a significant association between MIR181A2HG expression and the peritumoral MLVD (Figure 2D), thus suggesting MIR181A2HG as a promoter of lymphangiogenesis in vivo. However, HLECs co‐cultured with MIR181A2HG knockdown GC cells had no significant effect on lymphangiogenesis in vitro. This insinuates that factors beyond MIR181A2HG could contribute to GC‐induced lymphangiogenesis (Figure 2I; Figure S2A–C). Lymph node metastasis in tumors is a finely‐tuned, multi‐step process mediated by various factors. In addition to tumor cells inducing lymphangiogenesis, alterations in the invasive properties of tumor cells play a crucial role in promoting lymph node metastasis [29]. To further investigate the role of MIR181A2HG in GC invasiveness, we conducted transwell assays. Knocking down MIR181A2HG in SGC‐7901 and MKN‐45 cells significantly inhibited their invasion and migration (Figure S2D–G). These results collectively suggest that MIR181A2HG not only promotes lymphangiogenesis in GC but also enhances its invasiveness.

### 3.3 MIR181A2HG Induces M2 Macrophage Polarization to Mediate Lymphangiogenesis

**FIGURE 3:**
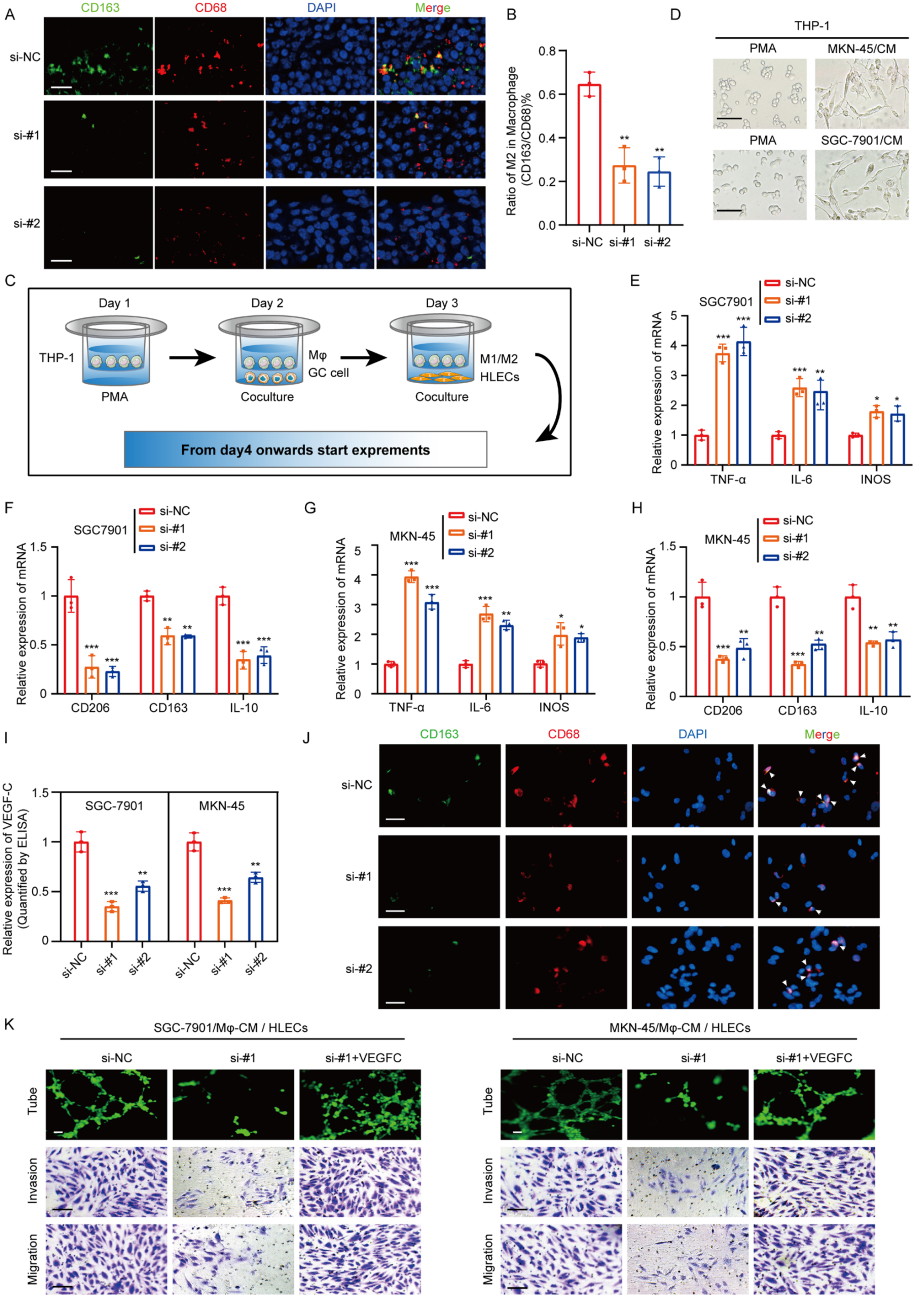
GC cell CM induces M2‐like polarization of macrophages, promoting lymphangiogenesis. (A) Immunofluorescence shows changes in the M2‐type macrophage marker (CD163) after treatment with conditioned medium from different treatment groups: Si‐NC (control group) and si‐#1 (si‐MIR181A2HG‐1) (scale bar, 50 μm). (B) Quantification of the Proportion of M2 Macrophages (CD163) Among Total Macrophages (CD68). (C) Schematic of the co‐culture model of GC cells and macrophages promoting HLECs lymphangiogenesis. (D) Typical images of macrophage morphological changes after treatment with PMA and GC cell CM (scale bar, 20 μm). (E–H) qRT‐PCR detection of typical M2 markers (CD163, CD206, and IL‐10) and M1 markers (iNOS, IL‐6, and TNFα) in PMA‐treated THP‐1 cells cultured with CM from different treatment groups (si‐NC, si‐#1 and si‐#2) of GC cells. (I) ELISA experiment detects the content of VEGF‐C in the CM after culturing macrophages with CM from different treatment groups (si‐NC, si‐#1 and si‐#2) of GC cells. (J) Immunofluorescence shows changes in the macrophage marker (CD163) after treatment with CM from different treatment groups: Si‐NC, si‐#1 and si‐#2 (scale bar, 50 μm). (K) Tube formation and transwell experiments detect the effects of tube formation and migration invasion ability of HLECS by macrophage CM from different treatment groups: Si‐NC, si‐#1 and si‐#1 + recombinant VEGF‐C protein. Statistical significance was assessed using two‐tailed *t*‐tests. *p < 0.05, **p < 0.01, ***p < 0.001 (scale bar, 50 μm).

Research indicates that due to changes in the microenvironment, macrophages can transform into M2 macrophages, which are closely associated with lymphangiogenesis. They can promote lymphatic vessel formation in LNs by producing VEGF‐C [30, 31]. To evaluate the effect of MIR181A2HG on macrophage M2 polarization, the CD163 (M2 macrophage marker) and CD68 (macrophage marker) were detected by immunofluorescence assay in the primary footpad tumors to analyze the M2 macrophage abundance. The results showed a decrease in M2 macrophage abundance in the si‐MIR181A2HG group compared to the si‐NC group (Figure 3A,B). Our qRT‐PCR results revealed that supernatant from si‐MIR181A2HG GC cells significantly decreased CD163, IL‐10, Arg‐1 (M2 macrophage markers) and VEGF‐C expression while increasing iNOS, IL‐6 and TNF‐α (M1 macrophage markers) expression in PMA‐treated THP‐1 cells compared to the control group (Figure 3C–I). The immunofluorescence results corroborated the findings, suggesting that MIR181A2HG in GC cells promotes M2 macrophage polarization (Figure 3J; Figure S3A). We then discovered that lymphatic endothelial cells treated with TAM derived CM exhibited significantly reduced tube formation, migration, and invasion capabilities in the si‐MIR181A2HG group compared to the NC (Figure 3K; Figure S3B–D). Meanwhile, we also co‐cultured THP‐1 cells or IL‐4‐induced M2 macrophages separately with HLECs. The results showed that M2 macrophages significantly promoted lymphangiogenesis, migration, and invasion of HLECs, whereas THP‐1 cells did not notably alter these processes (Figure S3E–G). These results suggest that MIR181A2HG fosters in vitro lymphangiogenesis by promoting M2 macrophage polarization. The addition of VEGF‐C recombinant protein to TAM‐derived medium significantly restored HLECs formation, migration, and invasion abilities (Figure 3K; Figure S3B–D). MIR181A2HG participates in lymphangiogenesis and lymphatic metastasis mainly by affecting M2 polarization of macrophages and VEGF‐C secretion.

### 3.4 MIR181A2HG Predominantly Localized in Cytoplasm and Acted as a Sponge for miRNA‐5680

**FIGURE 4:**
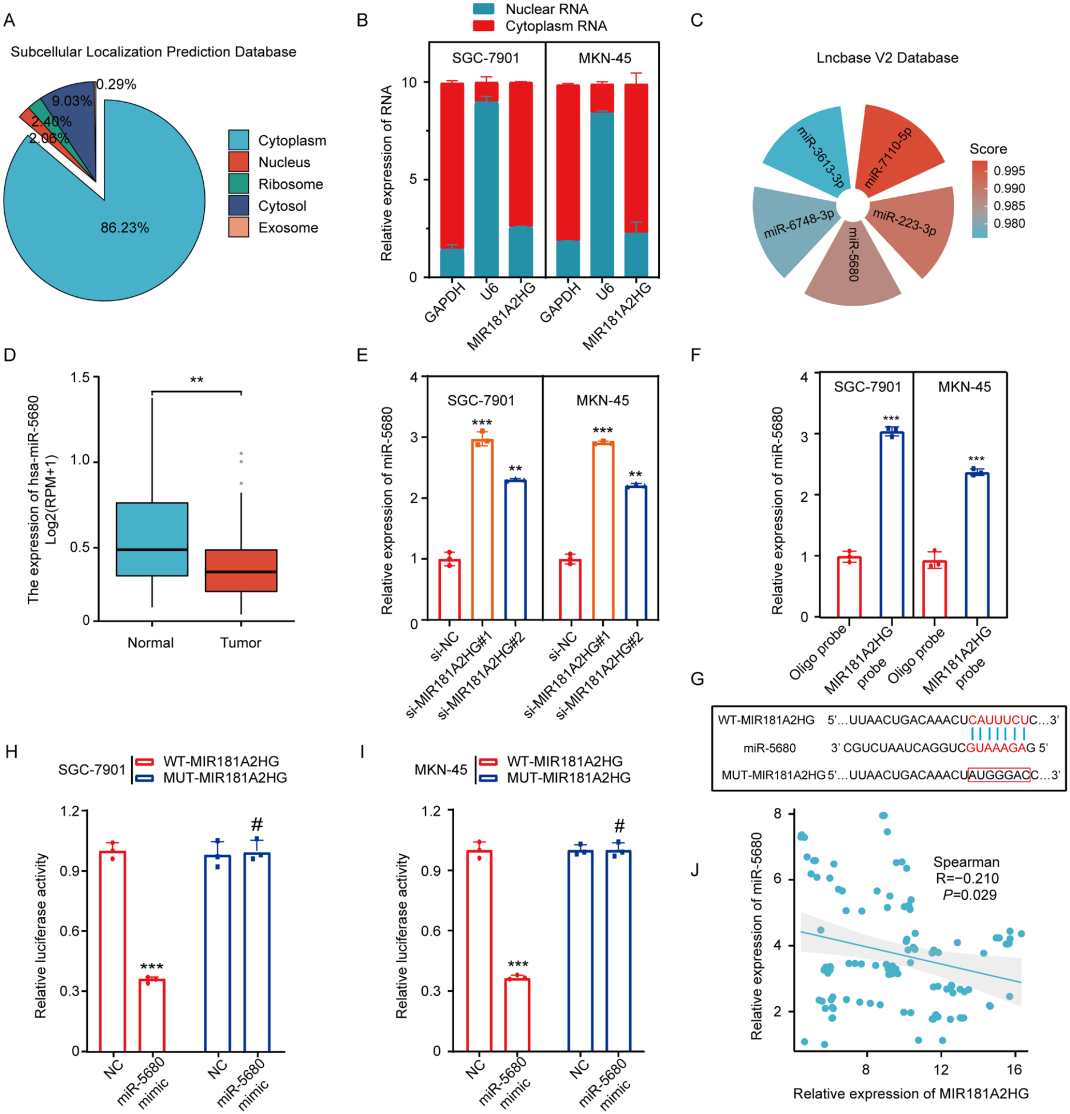
LncRNA MIR181A2HG predominantly localized in cytoplasm and acted as a sponge for miRNA‐5680. (A) Prediction of MIR181A2HG subcellular localization based on the online database subcellular localization prediction. (B) Nuclear separation analysis and qRT‐PCR analysis of MIR181A2HG expression in the nucleus and cytoplasm. U6 was used as a nuclear control, and GAPDH was used as a cytoplasmic control. (C) Prediction of downstream miRNA of MIR181A2HG based on the online database Lncbase. (D) Expression level of miR‐5680 in cancer and normal tissues in the TCGA STAD database. (E) qRT‐PCR detection of miR‐5680 expression in different treatment groups (si‐NC, si‐MIR181A2HG#1 and si‐MIR181A2HG#2) of GC cells. (F) RNA pulldown experiment detects the mutual binding of MIR181A2HG and miR‐5680. (G) Prediction of binding sites of MIR181A2HG and miR‐5680 based on the Lncbase database. (H, I) Luciferase reporter gene detection of MIR181A2HG wild type or binding site mutation (mutant type) in NC or miR‐5680 overexpressed cells. (J) Analysis of the correlation between MIR181A2HG and miR‐5680 expression based on qRT‐PCR detection results of 108 GC patient tissues. Statistical significance was assessed using two‐tailed *t*‐tests. **p < 0.01, ***p < 0.001, ^#^ *p* > 0.05.

LncRNAs typically exert their effects by interacting with downstream target miRNAs [14]. Using the subcellular localization prediction database lncLocator and a nuclear/cytoplasmic fractionation assay, we found that MIR181A2HG is primarily distributed in the cytoplasm (Figure 4A,B). The LncBase database predicted that miR‐7110‐5p, miR‐223‐3p, miR‐5680, miR‐6748‐3p, and miR‐3613‐3p have potential interactive sites with MIR181A2HG (Figure 4C). However, only miR‐5680 was underexpressed in GC patients in the TCGA database (Figure 4D; Figure S4A–D). qRT‐PCR analysis revealed that miR‐5680 is underexpressed in the GC cell lines compared to the gastric mucosa cell line (GES‐1) (Figure S4E). Meanwhile, qRT‐PCR validation revealed an inverse relationship between MIR181A2HG levels and miR‐5680 in GC cells (Figure 4E). Then, using a biotin‐labeled probe in an RNA pull‐down assay, we verified the significant enrichment of miR‐5680 over the control group, suggesting that MIR181A2HG directly interacts with miR‐5680 in GC cells (Figure 4F). Next, potential sequences of MIR181A2HG (WT) predicted to be complementary to miR‐5680's sequence were mutated (MUT) and used to construct a dual‐luciferase reporter vector (Figure 4G). The reporter assay showed that co‐transfecting MIR181A2HG‐WT and miR‐5680 significantly inhibited luciferase activity in comparison with the reference group. However, this suppression was absent when using the MIR181A2HG MUT variant (Figure 4H,I), indicating that WT is the core binding site for MIR181A2HG to sponge miR‐5680 in a ceRNA manner. Finally, there was also a negative correlation between the expression of MIR181A2HG and miR‐5680 in 108 GC tissues (Figure 4J). These results indicated that miR‐5680 participates in the mechanism underlying the functions of MIR181A2HG.

### 3.5 MIR181A2HG Modulates VCAN Expression Positively by Sponging miR‐5680

**FIGURE 5:**
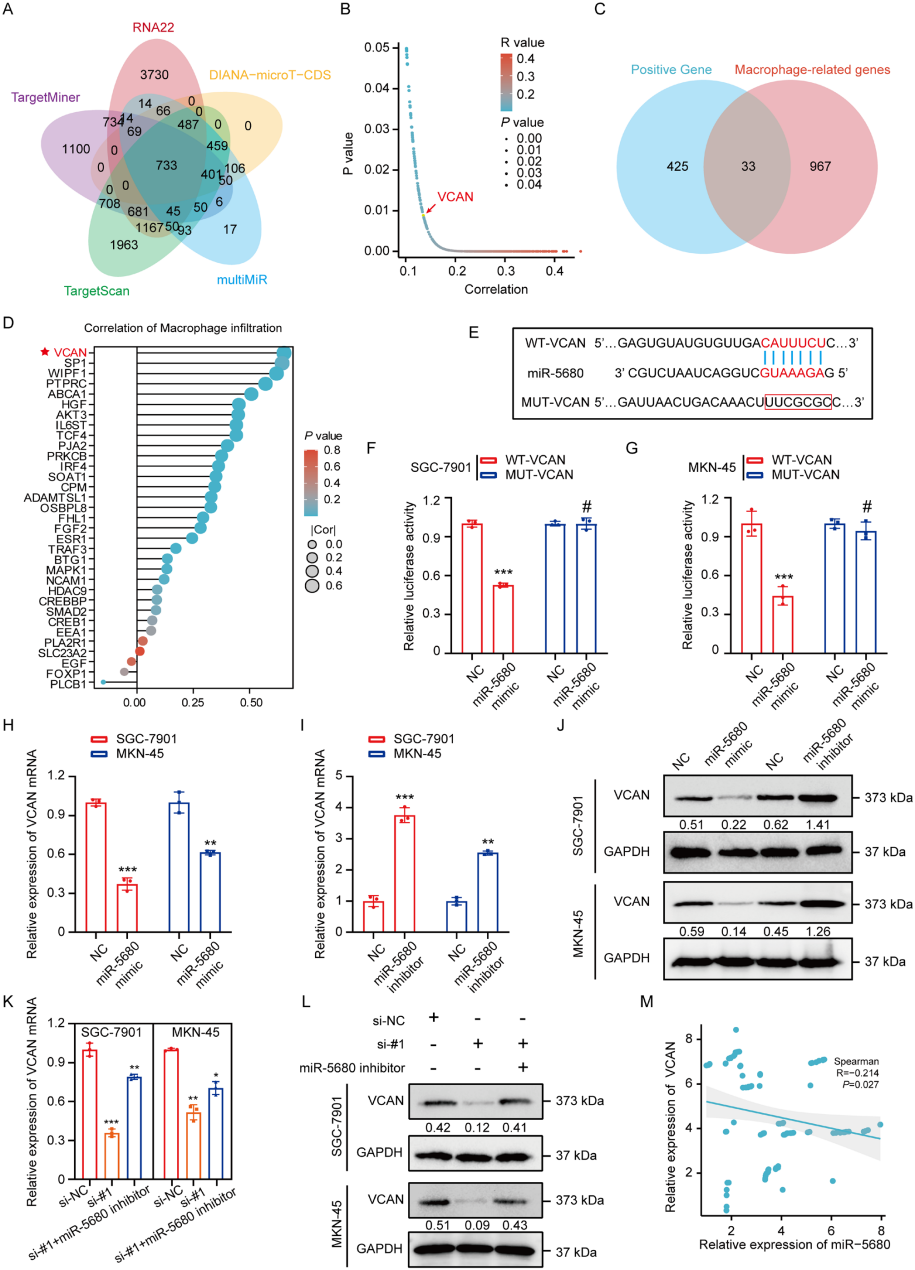
LncRNA MIR181A2HG modulates VCAN expression positively by sponging miR‐5680. (A) Prediction of downstream target genes of miR‐5680 based on online databases TargetMiner, RNA22, DIANA‐microT‐CDS, TargetScan, and MultiMIR. (B) In the TCGA database, 458 target genes are positively correlated with MIR181A2HG. (C) Venn diagram shows the intersection of 458 target genes positively correlated with MIR181A2HG and the top 1000 genes related to macrophages predicted by the GENEcard database. (D) Macrophage infiltration analysis for 33 target genes in TCGA STAD database. (E) Prediction of binding sites of miR‐5680 and VCAN based on the online database TargetScan. (F, G) Luciferase reporter gene detection of VCAN wild type or binding site mutation (mutant type) in NC or miR‐5680 overexpressed cells. (H–L) qRT‐PCR and WB detection of VCAN mRNA and protein expression levels in different treatment groups of GC cells: MiR‐NC, miR‐5680 mimic, miR‐5680 inhibitor, si‐NC, si‐#1 (si‐MIR181A2HG‐1), si‐#1 + miR‐5680. (M) Analysis of the correlation between VCAN and miR‐5680 expression based on qRT‐PCR detection results of 108 GC patient tissues. Statistical significance was assessed using two‐tailed *t*‐tests. *p < 0.05, **p < 0.01, ***p < 0.001, ^#^ *p* > 0.05.

To identify potential target genes for miR‐5680, we performed bioinformatic analyses using the TargetMiner, RNA22, DIANA‐microT‐CDS, TargetScan and MultiMIR databases, which together predicted 733 target genes (Figure 5A). Of these, 458 were positively correlated with MIR181A2HG in the TCGA database (Figure 5B). The intersection of 458 target genes with macrophage‐related genes from Genecards top1000 revealed 33 genes (Figure 5C). Finally, these 33 genes were analyzed for macrophage‐related immune infiltration, and it was found that VCAN ranked first, so we chose VCAN for further study (Figure 5D). To corroborate the association between miR‐5680 and VCAN within GC cells, we conducted a luciferase experiment. This confirmed that the co‐transfection with miR‐5680 mimic notably impeded the activity of the WT VCAN luciferase reporter construct, with no such effect on the MUT variant (Figure 5E–G). Furthermore, qPCR and Western blot assays showed that miR‐5680 overexpression down‐regulated VCAN expression, while miR‐5680 knockdown up‐regulated VCAN expression in GC cells (Figure 5H–J). In GC cells with si‐MIR181A2HG transfection, there was a noticeable reduction in VCAN mRNA and protein levels. In contrast, transfection with the miR‐5680 inhibitor displayed an elevation in these levels (Figure 5K,L). Furthermore, a discernible inverse relationship between VCAN and miR‐5680 was observed across 108 GC tissue specimens (Figure 5M). This data suggests that in GC, VCAN is targeted by miR‐5680.

### 3.6 The MIR181A2HG/miR‐5680/VCAN Axis Affects Lymphangiogenesis by Influencing the Polarization of M2‐Type Macrophages

**FIGURE 6:**
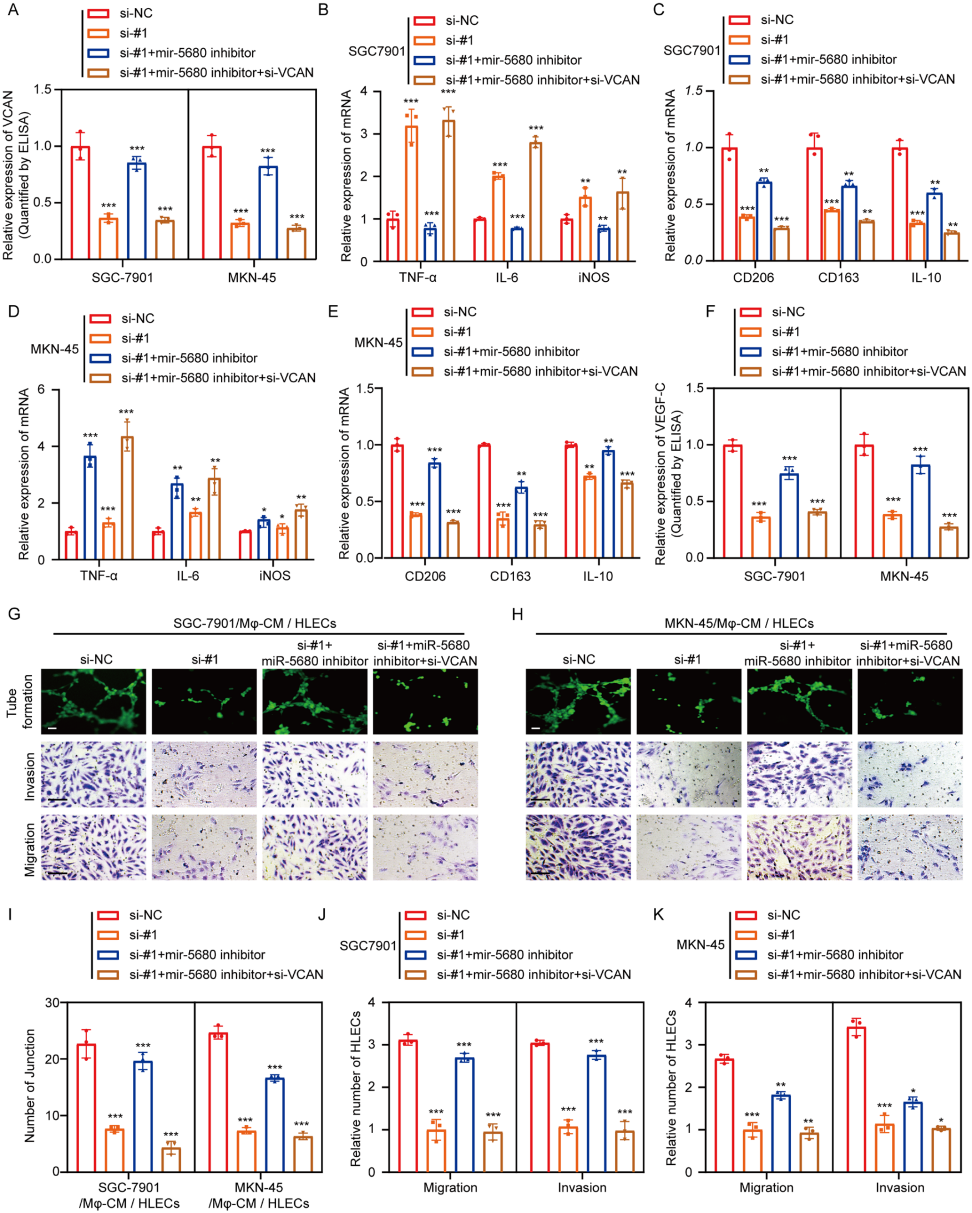
The MIR181A2HG/miR‐5680/VCAN axis affects lymphangiogenesis by influencing the polarization of M2‐type macrophages. (A) Elisa detection of VCAN protein expression levels in different treatment groups of GC cells: NC, si‐#1 (si‐MIR181A2HG‐1), si‐#1 + miR‐5680 inhibitor, si‐#1 + miR‐5680 inhibitor +si‐VCAN. (B–E) qRT‐PCR detection of typical M2 markers (CD163, CD206, and IL‐10) and M1 markers (iNOS, IL‐6, and TNFα) in PMA‐treated THP‐1 cells cultured with CM from different treatment groups (NC, si‐#1 (si‐MIR181A2HG‐1), si‐#1 + miR‐5680 inhibitor, si‐#1 + miR‐5680 inhibitor +si‐VCAN) of GC cells. (F) ELISA experiment detects the content of VEGF‐C in the supernatant after culturing macrophages with CM from different treatment groups (NC, si‐#1 (si‐MIR181A2HG‐1), si‐#1 + miR‐5680 inhibitor, si‐#1 + miR‐5680 inhibitor +si‐VCAN) of GC cells. (G–K) Tube formation and transwell experiments detect the effects and quantification of tube formation and migration invasion ability of HLECS by macrophage CM from different treatment groups: NC, si‐#1 (si‐MIR181A2HG‐1), si‐#1 + miR‐5680 inhibitor, si‐#1 + miR‐5680 inhibitor +si‐VCAN. Statistical significance was assessed using two‐tailed *t*‐tests. *p < 0.05, **p < 0.01, ***p < 0.001 (scale bar, 50 μm).

Following this, we embarked on compensatory trials aiming to elucidate the nexus between miR‐5680, VCAN, and the capacity of MIR181A2HG to modulate M2 macrophage orientation. Upon transfection with miR‐5680 suppressors, the impact of MIR181A2HG depletion on macrophage orientation was mitigated. However, this compensatory effect was nullified once VCAN underwent silencing (Figure 6A–E; Figure S5A,B). The results of lymphatic regeneration, migration, invasion and VEGF‐C expression were also verified (Figure 6F–K). Taken together, these data suggest that the miR‐5680/VCAN axis is an important mediator for MIR181A2HG to regulate the polarization of M2‐type macrophages and thus further affect lymphangiogenesis.

### 3.7 VCAN Induces M2 Macrophage Activation via Binding to CD44

**FIGURE 7:**
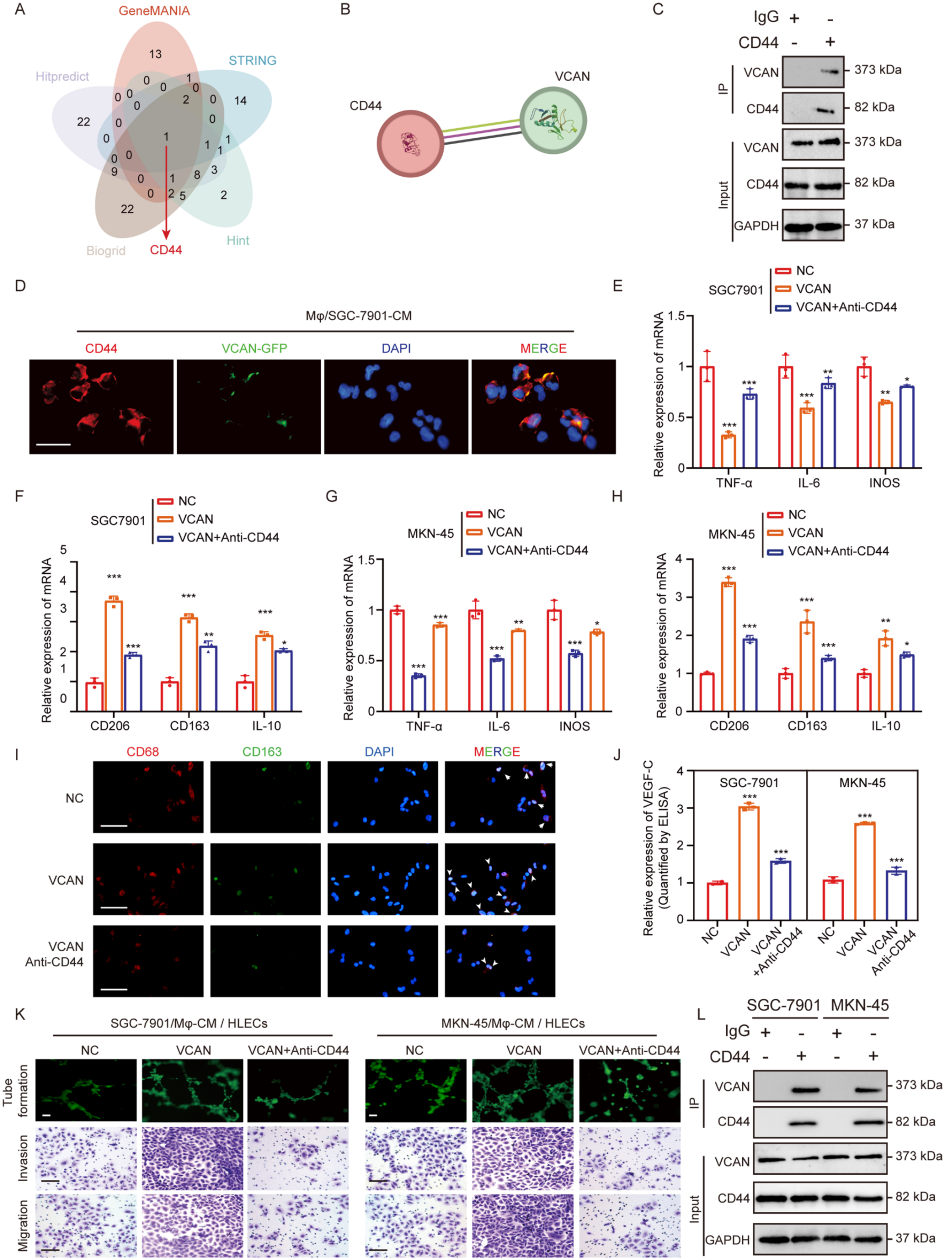
VCAN Induces M2 Macrophage Activation via Binding to CD44. (A) Prediction of VCAN interacting proteins based on online databases String, GeneMANIA, Hitpredict, Biogrid, Hint. (B) String database predicts CD44 interacts with VCAN. Purple line: Experimentally determined. Green line: Text mining. Black line: Co‐expression. (C) Co‐IP experiment detects the mutual binding of CD44 and VCAN in polarized macrophages. (D) Immunofluorescence detects the co‐localization of CD44 and VCAN in polarized macrophages (scale bar, 50 μm). (E–H) qRT‐PCR detection of typical M2 markers (CD163, CD206, and IL‐10) and M1 markers (iNOS, IL‐6, and TNFα) in PMA‐treated THP‐1 cells cultured with CM from different treatment groups (NC, VCAN recombinant protein, VCAN recombinant protein + anti‐CD44) of GC cells. (I) Immunofluorescence shows changes in the macrophage marker (CD163) after treatment with CM from different treatment groups: NC, VCAN recombinant protein, VCAN recombinant protein + anti‐CD44 (scale bar, 50 μm). (J) ELISA experiment detects the content of VEGF‐C in the supernatant after culturing macrophages with CM from different treatment groups (NC, VCAN recombinant protein, VCAN recombinant protein + anti‐CD44) of GC cells. (K) Tube formation and transwell experiments detect the effects of macrophage CM from different treatment groups (NC, VCAN recombinant protein, VCAN recombinant protein + anti‐CD44) on the tube formation and migration invasion ability of HLECS (scale bar, 50 μm). (L) Co‐IP experiment detects the mutual binding of CD44 and VCAN in GC cells. *p < 0.05, **p < 0.01, ***p < 0.001.

Based on our previous findings, we aimed to investigate the mechanism of VCAN in activating TAMs. Through the String, GeneMANIA, Hitpredict, Biogrid, Hint database, we predicted the VCAN interacting protein CD44 (Figure 7A,B). Previous studies have shown that VCAN in the extracellular matrix can bind specifically to the cell membrane receptor CD44, thereby activating downstream pathways and altering cellular behaviors [32, 33]. We validated this interaction through Co‐IP and IF co‐localization experiments (Figure 7C,D). qRT‐PCR results showed that macrophages of M2 type increased significantly when cultured with VCAN recombinant protein or the addition of CD44 neutralizing antibody (anti‐CD44) could reverse this process (Figure 7E–I; Figure S5C). Furthermore, the secretion of VEGF‐C from macrophages and its effects on lymphangiogenesis and the migration and invasion ability of HLECs were also consistent with this trend (Figure 7J,K; Figure S5D–F). Interestingly, since the CD44 receptor is also present on the surface of GC cells, we confirmed through Co‐IP experiments that VCAN can bind to CD44 (Figure 7L).

### 3.8 MIR181A2HG/miR‐5680/VCAN/CD44/SP1 Forms a Positive Feedback Loop

**FIGURE 8:**
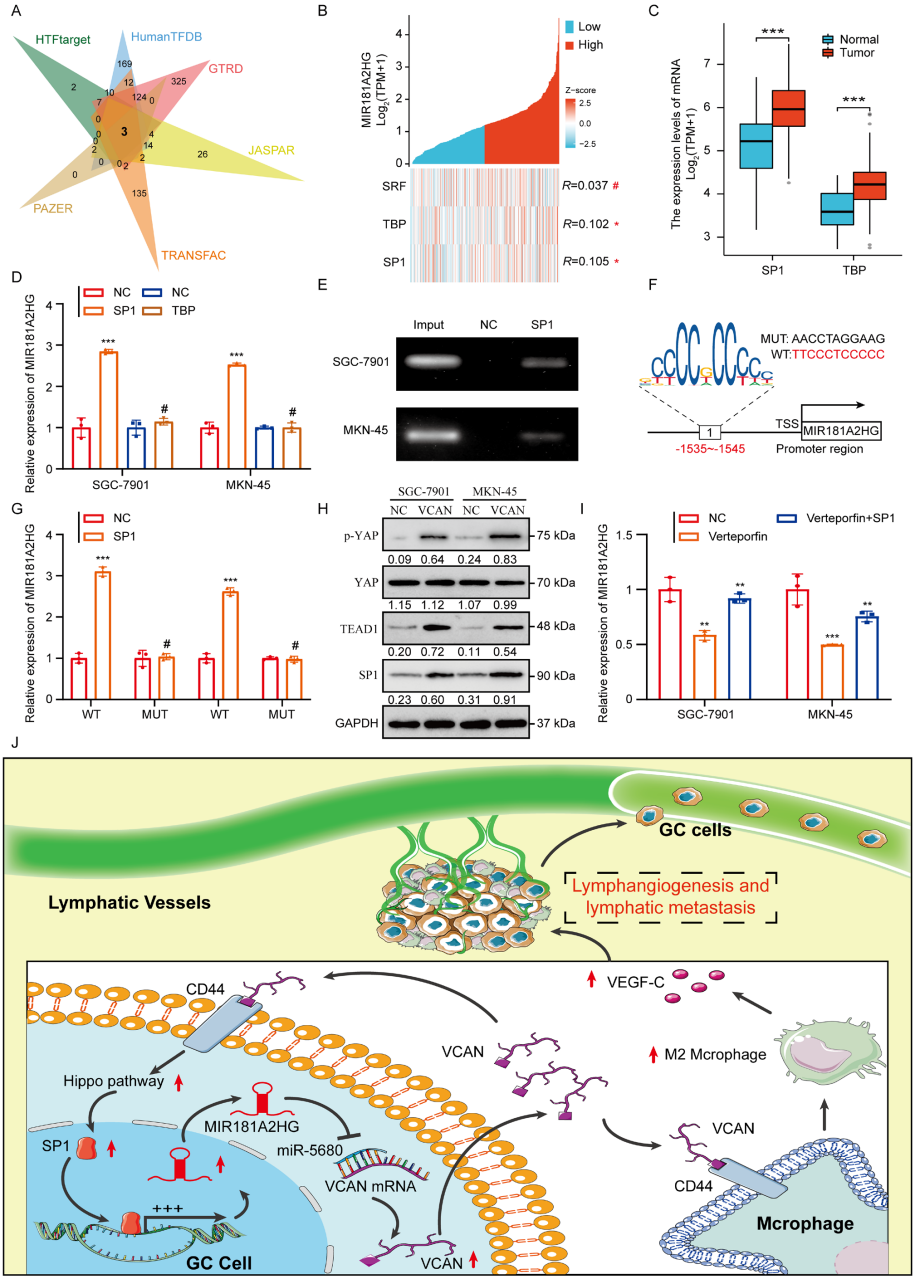
MIR181A2HG/miR‐5680/VCAN/CD44/SP1 forms a positive feedback loop. (A) Prediction of upstream transcription factors of MIR181A2HG based on online databases Human TFDB, PAZAR, GTRD, TRANSFAC, HTFtarget, and JASPAR. (B) Correlation analysis of three transcription factors predicted in the TCGA STAD database: SRF, TBP, SP1 with MIR181A2HG. (C) Expression levels of SP1 and TBP in the TCGA STAD database. (D) qRT‐PCR analysis of MIR181A2HG in GC cells after SP1 and TBP overexpression. (E) The direct binding relationship between SP1 and MIR181A2HG was demonstrated by ChIP assay. (F) The potential binding sites of SP1 in the MIR181A2HG promoter region based on analysis of JASPAR database. (G) Luciferase reporter assay of MIR181A2HG WT and Mut after transfection of the SP1 plasmid. (H) Western blot detected the protein level of the Hippo pathway after application of recombinant VCAN protein. (I) qRT‐PCR analysis of MIR181A2HG expression in GC cells with SP1 overexpression with or without Hippo pathway inhibitor (Verteporfin). (J) Schematic representation of the positive feedback loop. *p < 0.01, ***p < 0.001, ^#^ *p* > 0.05.

To delve into the molecular underpinnings behind the elevated levels of MIR181A2HG in GC, it is of great significance to explore the interaction between transcription factors and lncRNAs promoter regions, aiming to unravel the complexity of gene regulatory networks within cells and understand the functionality of lncRNAs [34]. By intersecting candidate transcription factors for MIR181A2HG from the Human TFDB, PAZAR, GTRD, TRANSFAC, HTFtarget, and JASPAR databases, SP1, TBP (TATA‐Box Binding Protein) and SRF (Serum Response Factor) was identified as the transcription factor common to all databases, capable of binding to the MIR181A2HG promoter (Figure 8A). Analysis of the TCGA STAD dataset revealed that SP1 and TBP were positively correlated with MIR181A2HG, and both were highly expressed in GC tissues (Figure 8B,C). Overexpression of SP1 increased MIR181A2HG expression in GC cells, but TBP had no significant effect (Figure 8D). ChIP experiments further confirmed the binding of SP1 to the MIR181A2HG promoter (Figure 8E). The dual‐luciferase reporter assays showed that overexpressing SP1 amplified the luciferase activity steered by the MIR181A2HG promoter. However, upon mutating the putative SP1 binding site within the MIR181A2HG promoter, SP1 overexpression no longer influenced the luciferase activity from the altered promoter (Figure 8F,G). This suggests that SP1 physically associates with the MIR181A2HG promoter, amplifying its transcription in GC cells. Notably, previous studies have identified SP1 as a target downstream of the CD44/Hippo signaling cascade [35]. Western blot analysis confirmed that overexpression of VCAN significantly increased the level of SP1 protein in GC cells, and inhibitors of the Verteporfin hippo pathway could reverse this process (Figure 8H). qRT‐PCR results showed that Verteporfin could significantly inhibit the expression level of MIR181A2HG, while overexpression of SP1 could restore this process in GC cells (Figure 8I). Consequently, we uncovered a positive regulatory feedback loop shaped by the MIR181A2HG/miR‐5680/VCAN/CD44/SP1 axis, leading to sustained stimulation of MIR181A2HG expression.

## 4 Discussion

Lymph node (LN) metastasis is associated with a poor prognosis in gastric cancer patients and currently lacks effective treatment options in clinical settings. Therefore, exploring the molecular mechanisms driving LN metastasis and identifying novel, promising targets for prevention and therapy are of critical importance (PMID: 37158903). Tumor microenvironment‐induced VEGF‐C is pivotal in promoting lymphangiogenesis, a key limiting factor for LN metastasis in cancer (PMID: 38847478). However, the detailed mechanisms remain unclear, particularly regarding how VEGF‐C is regulated within the tumor microenvironment. In this study, we identified the long non‐coding RNA MIR181A2HG, which is overexpressed in gastric cancer and is associated with poor prognosis. It drives M2 polarization of TAMs, thereby promoting lymphangiogenesis. Mechanistically, we revealed that the MIR181A2HG/miR‐5680 axis functions as a competing endogenous RNA regulatory network to upregulate VCAN. On one hand, VCAN interacts with CD44 receptors on TAMs via paracrine signaling, inducing M2 macrophage polarization and increasing VEGF‐C secretion, which enhances lymphangiogenesis. On the other hand, VCAN binds to CD44 receptors on GC cells via autocrine signaling, activating the Hippo pathway and upregulating SP1, leading to increased transcription of MIR181A2HG. This creates a feedback loop that drives lymphatic metastasis. The progression of cancer metastasis occurs over time and across different locations. A favorable local environment is crucial for circulating tumor cells to settle and proliferate in remote areas [36]. Macrophages are pivotal in creating this environment [37]. In a related study, Wei Li et al. reported that macrophages induced by gastric cancer cells significantly promote metastasis by facilitating the EMT in these cells [38]. Linde et al. observed that depleting macrophages inhibits early breast cancer metastasis and reduces the metastatic burden in the lungs during the later stages of cancer development [39]. Yet, the exact molecular pathways through which macrophages facilitate metastasis are still not clear. Macrophages can be classified into immunogenic M1 macrophages and the “alternatively activated” or “repair” M2 macrophages, based on their ability to engulf particles and their cytokine production patterns [40]. In our study, we specifically investigated the impact of VCAN from GC on macrophage phenotype changes. Using qRT‐PCR, immunofluorescence, and macrophage polarization markers, we found that VCAN originating from GC strongly induces the hepatic macrophage shift towards an M2 phenotype. It was observed that M2 polarized macrophages promote lymphangiogenesis by secreting VEGF‐C. Nevertheless, the molecular mechanisms instigating M2 polarization in GC macrophages require further investigation.

Moreover, an essential discovery was that MIR181A2HG upregulates VCAN expression by competitively binding to miR‐5680. VCAN, exhibiting elevated expression and secretion in tumor cells, advances cancer development [41]. VCAN modulates the TME by encouraging TAM infiltration through binding with CD44 [32]. CD44 stimulates cancer progression through the hippo pathway [42]. Thus, elucidating the precise molecular mechanisms behind the persistent expression of VCAN in GC can provide potential predictive factors for effective anti‐VCAN therapies. In this research, we demonstrated that VCAN not only promotes M2 polarization of macrophages but also binds to the CD44 receptor on the surface of GC cells. This establishes a feedback loop, continually stimulating the upregulation of MIR181A2HG. However, the specific molecular mechanisms behind the mutual binding of VCAN and CD44 still need exploration.

MIR181A2HG has been studied in various cancers. Wang et al. found that the downregulation of MIR181A2HG disturbs endothelial cell glucose metabolism, leading to endothelial cell damage in hyperglycemic conditions [43]. Wu et al. and Su et al. proved, through bioinformatics analysis and cellular experiments, that MIR181A2HG is associated with poor prognosis in bladder and thyroid cancers [44, 45]. There have been no studies related to GC, but our research filtered out the high expression of MIR181A2HG in GC. We demonstrated that silencing MIR181A2HG plays a crucial role in suppressing tumor lymphatic metastasis. Recently, drugs designed based on lncRNA has demonstrated notable anti‐cancer properties in vivo, selectively targeting tumor cells without impacting healthy cells. This suggests that MIR181A2HG might be a target for cancer therapy, but how exactly to design it requires further research.

## 5 Conclusion

In conclusion, our study provides compelling evidence that MIR181A2HG overexpression is clinically and functionally linked to lymphatic metastasis in human gastric cancer through the MIR181A2HG/miR‐5680/VCAN‐CD44 axis. This axis modulates the tumor microenvironment by regulating VEGF‐C secretion in a TAMs M2 macrophage polarization‐dependent manner. Elucidating the role of MIR181A2HG in promoting LN metastasis and activating M2 macrophage polarization in TAMs enhances our understanding of lncRNA‐driven metastasis and offers potential avenues for developing therapeutic strategies targeting lymph node metastasis in gastric cancer.

## Author Contributions

**Weijie Zang:** writing – original draft (equal). **Yongpu Yang:** investigation (equal), methodology (equal). **Junjie Chen:** writing – review and editing (equal). **Qinsheng Mao:** methodology (equal). **Wanjiang Xue:** writing – review and editing (equal). **Yilin Hu:** data curation (equal), investigation (equal).

## Ethics Statement

This study was approved by the Human Research Ethics Committee of the Affiliated Hospital of Nantong University (Nantong, China, approval 2020‐L145), and each patient provided written informed consent. The study is compliant with all relevant ethical regulations involving human participants. Animal experiments were approved by Animal Center of Medical College of Nantong University.

## Consent

The authors have nothing to report.

## Conflicts of Interest

The authors declare no conflicts of interest.

## Supporting information


Figure S1.

Figure S2.

Figure S3.

Figure S4.

Figure S5.

Data S1.

## Data Availability

The data that support the findings of this study are available on request from the corresponding author.
