# Supplementary figures and images for: The MIR181A2HG/miR‐5680/VCAN‐CD44 Axis Regulates Gastric Cancer Lymph Node Metastasis by Promoting M2 Macrophage Polarization
Source: Cancer Med. 2025 Jan 16;14(2):e70600. doi: 10.1002/cam4.70600 (PMC11739459; doi:10.1002/cam4.70600)

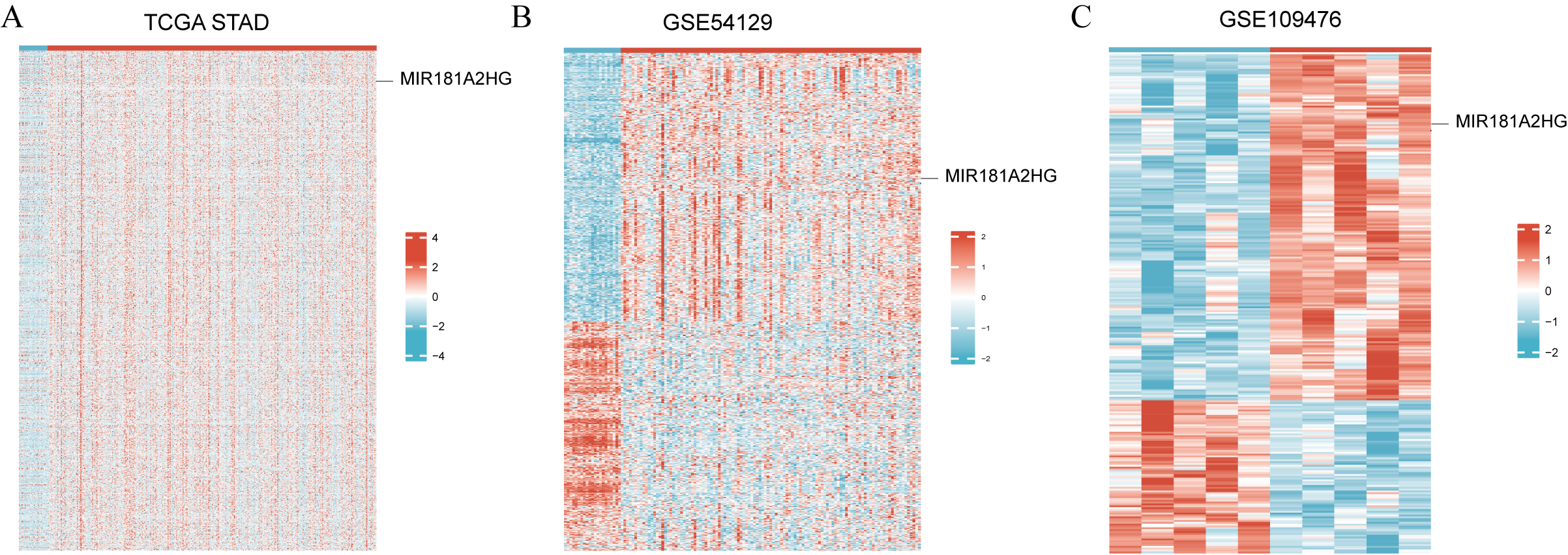

Supplement: Supplementary file 1 — Figure S1. [file CAM4-14-e70600-s004.tif]

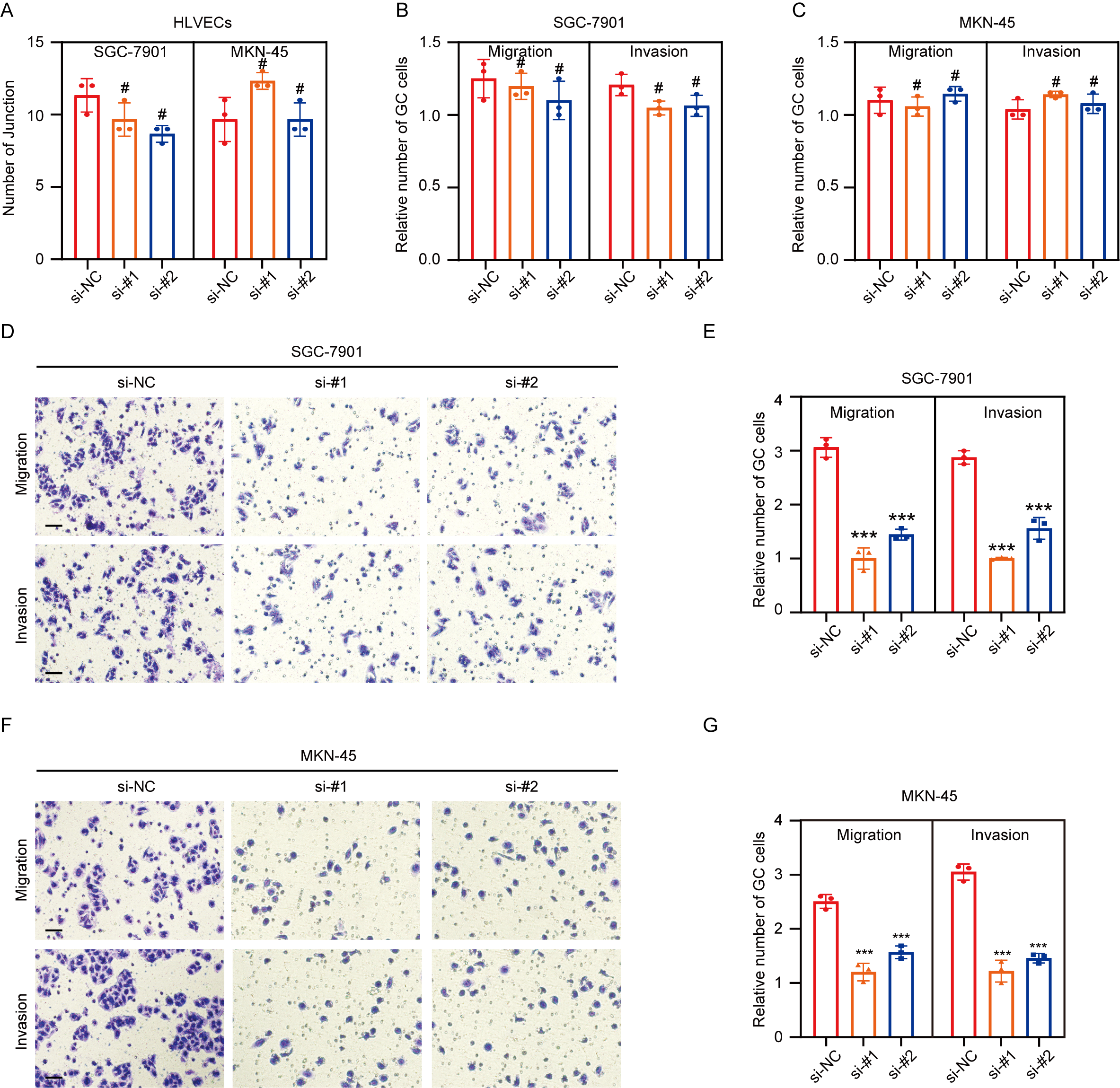

Supplement: Supplementary file 2 — Figure S2. [file CAM4-14-e70600-s005.tif]

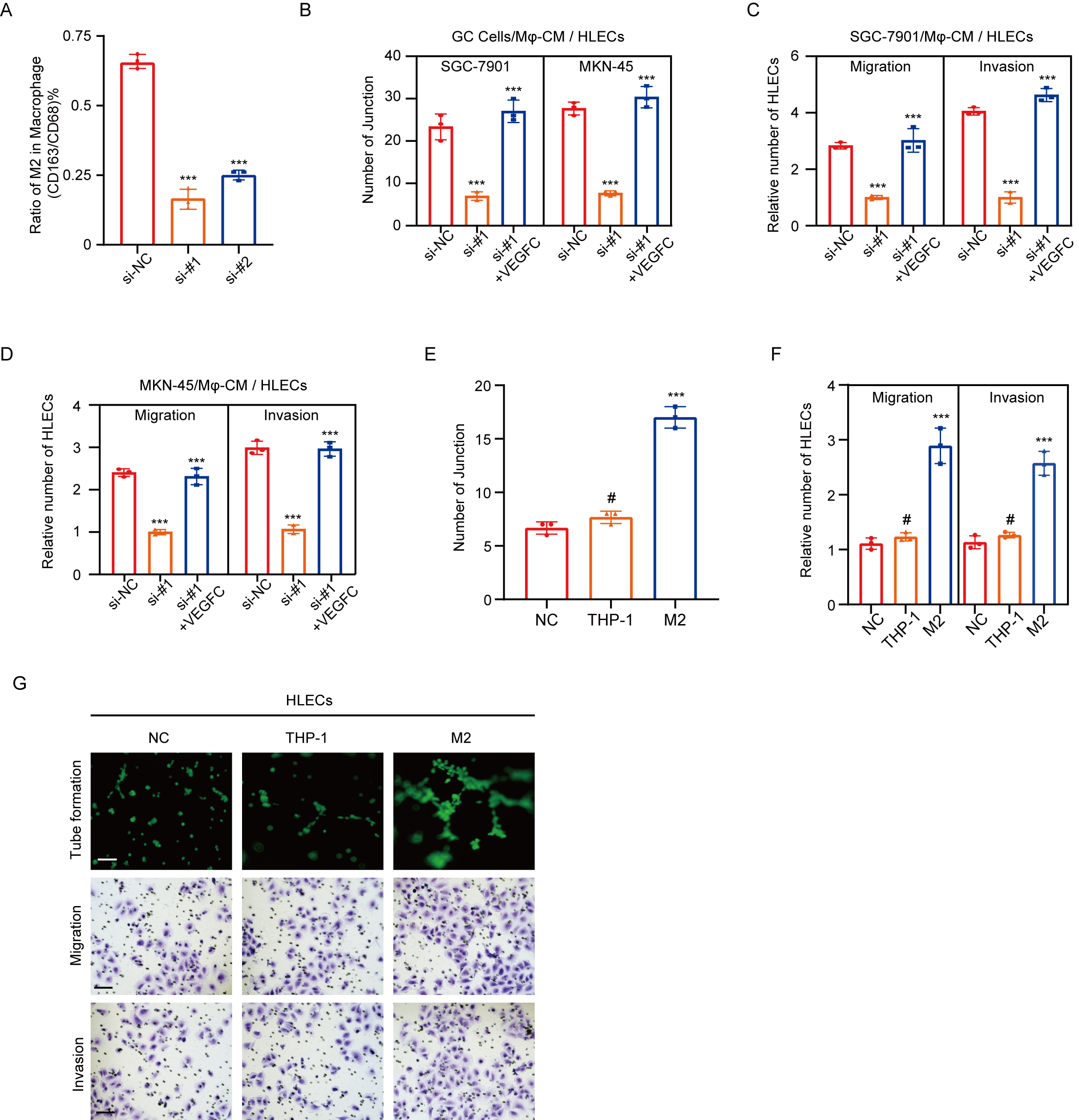

Supplement: Supplementary file 3 — Figure S3. [file CAM4-14-e70600-s001.tif]

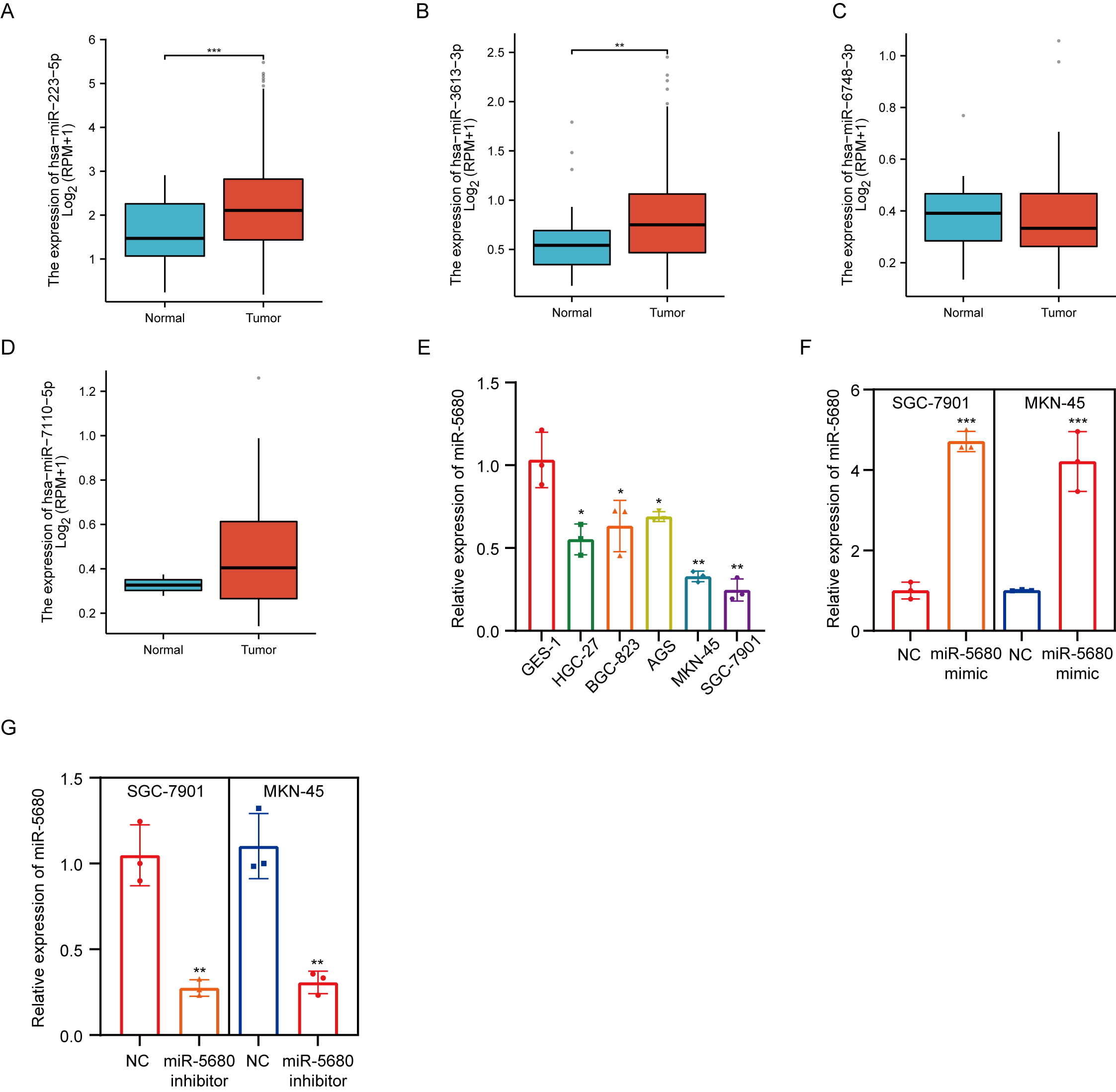

Supplement: Supplementary file 4 — Figure S4. [file CAM4-14-e70600-s002.tif]

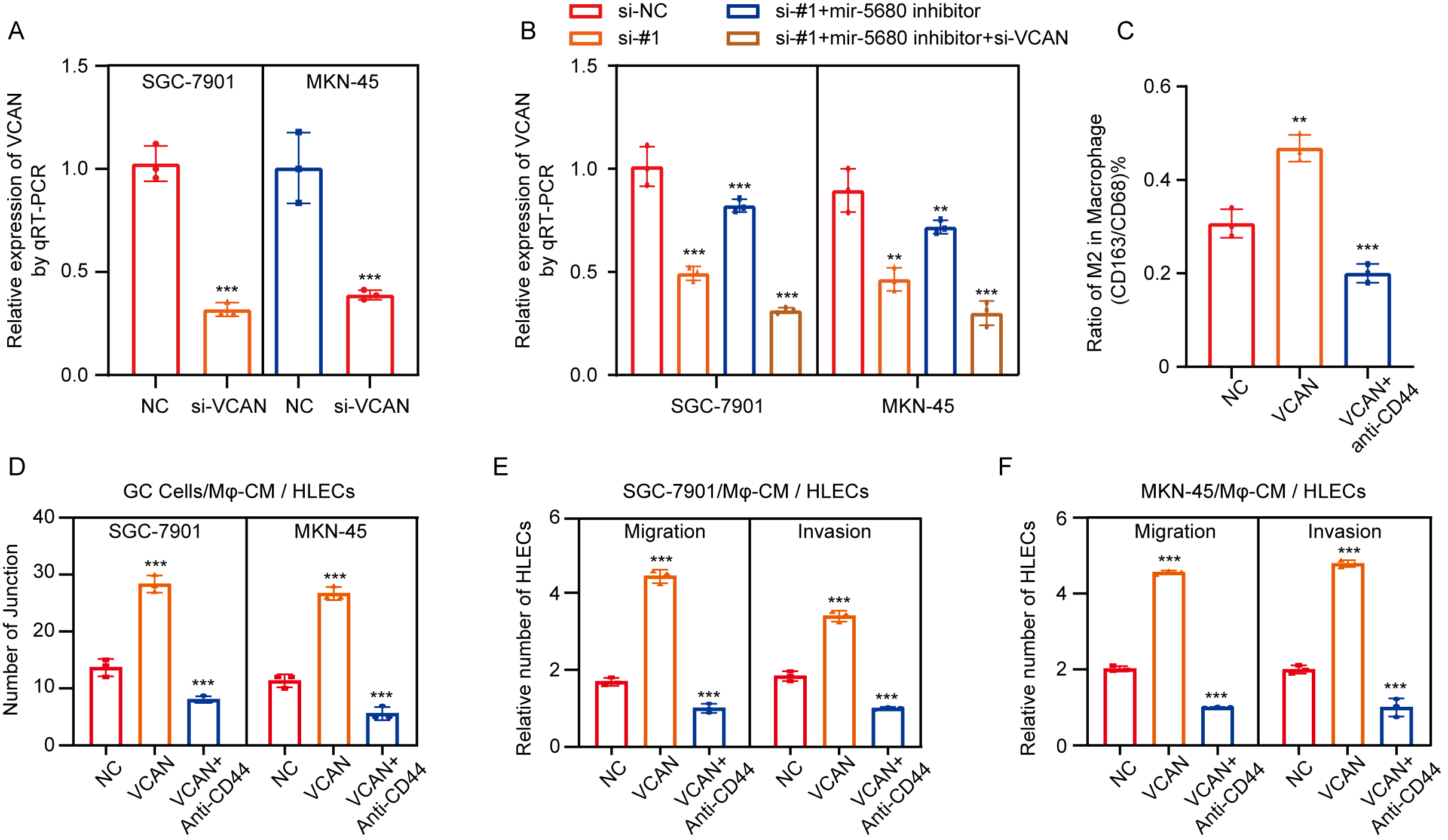

Supplement: Supplementary file 5 — Figure S5. [file CAM4-14-e70600-s006.tif]
