# Supplementary material for: The MIR181A2HG/miR‐5680/VCAN‐CD44 Axis Regulates Gastric Cancer Lymph Node Metastasis by Promoting M2 Macrophage Polarization
Source: Cancer Med. 2025 Jan 16;14(2):e70600. doi: 10.1002/cam4.70600 (PMC11739459; doi:10.1002/cam4.70600)
Supplement: Supplementary file 6 — Data S1. [file CAM4-14-e70600-s003.docx]

**Supplementary figure legends**

**Figure S1.** **Heatmap of Differentially Expressed Genes in Gastric Cancer Datasets.** A-B. Heatmap showing the expression of differentially expressed genes in TCGA STAD (A) and GEO (GSE54129 (B) and GSE109476 (C)) databases.

**Figure S2.** **Quantification of Tube Formation and Transwell Assays in HLECs and GC Cells Treated with siRNAs.** A-C. Quantification of Tube formation (A) and transwell (B and C) of HLECs treated with conditioned medium from different treatment groups: si-NC (control group), si-#1 (si-MIR181A2HG-1), si-#2 (si-MIR181A2HG-2). D-G. Transwell experiments detect the effects of migration and invasion ability of GC cells by si-#1 and si-#2. #*P* > 0.05, ****P* < 0.001 (scale bar, 50 μm).

**Figure S3.** **Quantification of M2 Macrophage Proportion and Impact of VEGF-C on Tube Formation and Migration in HLECs.**

**A.** Quantification of the Proportion of M2 Macrophages (CD163) Among Total Macrophages (CD68) in Fig. 3J. B-D. Quantification of Tube formation (A) and transwell (B and C) of HLECs treated with conditioned medium from different treatment groups: si-NC (control group), si-#1 (si-MIR181A2HG-1), si-#1+VEGF-C. E-F. Quantification of tube formation (E) and transwell (F) of HLECs corresponds to Figure G. G. Transwell and Tube formation experiments detect the effects of lymphangiogenesis, migration, and invasion ability of HLECs cells by NC (control group), THP-1 and M2 macrophage. #P > 0.05, ****P* < 0.001 (scale bar, 100 μm).

**Figure S4.** **miR-5680 Expression Levels in Gastric Cancer from TCGA STAD and GC Cells.** A-D. mRNA expression levels of miR-7110-5p, miR-223-3p, miR-6748-3p, and miR-3613-3p in the TCGA STAD database. E. qRT-PCR detection of miR-5680 expression in different GC cells and normal gastric mucosal epithelial cells. **P* < 0.05, ***P* < 0.01.

**Figure S5. Effect of VCAN and CD44 Neutralizing Antibody on HLEC Angiogenesis and Migration.** A. Relative expression of VCAN by qRT-PCR in SGC-7901 and MKN-45 cells after treatment with NC or si-VCAN. B. Relative expression of VCAN by qRT-PCR in SGC-7901 and MKN-45 cells after treatment with si-NC, si-#1, si-#1 + mir-5680 inhibitor, or si-#1 + mir-5680 inhibitor + si-VCAN. C. Quantification of the Proportion of M2 Macrophages (CD163) Among Total Macrophages (CD68) in Fig. 7I. D-F. Quantitative analysis of tube formation and transwell assays evaluating the impact of CM from macrophages subjected to various treatments: NC, VCAN recombinant protein, and VCAN recombinant protein + CD44 neutralizing antibody, on the angiogenic and migratory/invasive capabilities of HLECs in Fig 7K.

**Table S1. Target sequences**

| **Name** | **Target Seq** |
| --- | --- |
| **si-MIR181A2HG#1** | S: 5’-GAUGCAGAAUCUACCUACATT-3’  AS: 5’-UGUAGGUAGAUUCUGCAUCTT-3’ |
| **si-MIR181A2HG#2** | S: 5’-GCAUGGAGUAGAUAAUAAATT-3’  AS: 5’- UUUAUUAUCUACUCCAUGCTT-3’ |
| **si-MIR181A2HG#3** | S: 5’-CUGGGUUCCAGUAUCUAAUTT-3’  AS: 5’-AUUAGAUACUGGAACCCAGTT-3’ |
| **Si-NC** | S: 5’-UUCUCCGAACGUGUCACGUTT-3’  AS: 5’-ACGUGACACGUUCGGAGAATT-3’ |
| **has-miR-5680 inhibitor** | 5’-GCAGAUUAGUCCAGCAUUUCUC-3’ |
| **miRNA inhibitor NC** | 5’-CAGUACUUUUGUGUAGUACAA-3’ |
| **has-miR-5680 mimics** | 5’-GAGAAAUGCUGGACUAAUCUGC-3’  5’-AGAUUAGUCCAGCAUUUCUCUU-3’ |
| **miRNA mimic NC** | 5’-UUCUCCGAACGUGUCACGUTT-3’ |
| **si-VCAN** | S: 5′-GAGGCUGGAACUGUUAUUATT-3′  AS: 5′-UAAUAACAGUUCCAGCCUCTT-3′ |

**Table S2. Sequences of primers used for amplification of target genes**

| **Gene**  **primer nucleotide sequence** |
| --- |
| **MIR181A2HG** Forward: 5′- CGCGGTTCAATACCTCGTCT -3′  Reverse: 5′- TGCTGTGGCTAGAGGACAAC -3′  **TNF-α** Forward: 5′- - GTAGCCCACGTCGTAGCAAA -3′  Reverse: 5′- CCCTTCTCCAGCTGGGAGAC -3′  **IL-6**  Forward: 5′- TCTTGGGAC-TGATGCTGGTG -3′  Reverse: 5′- CAGAATTGCCATTGCACAACTC -3′  **INOS** Forward: 5′- CTGCAGCACTTGGATCAGGAACCTG -3′  Reverse: 5′- GGAGTAGCCTGTGTGCACCTGGAA-3′  **CD206**  Forward: 5′- AGTTGGGTTCTCCTGTAGCCCAA -3′  Reverse: 5′- ACTACTACCTGAGCCCACACCTGCT -3′  **CD163**  Forward: 5′- CGGACTTCTCTCTGGAAGC -3′  Reverse: 5′- CTCATGTCCCTCACACTGG -3′  **IL-10** Forward: 5′- GACTTTAAGGGTTACCTGGGTTG -3′  Reverse: 5′- TCACATGCGCCTTGATGTCTG -3′  **miR-5680**  Forward: 5′- GGCGAACTAGAGAAATGCTGGA -3′  Reverse: 5′- TATGGTTGTTCACGACTCCTTCAC-3′  **VCAN**  Forward: 5′- GAACCCTGTATCGTTTTGAGAACC -3′  Reverse: 5′- CAGCCCCCAGCAAGCACAAAATT -3′  **GAPDH**  Forward: 5′- GAGTCAACGGATTTGGTCGT -3′  Reverse: 5′- TGGGTGGAATCATATTGGAA -3′  **U6**  Forward: 5′- ATTGGAACGATACAGAGAAGATT -3′  Reverse: 5′- GGAACGCTTCACGAATTT G -3  **SP1**  Forward: 5′- GGTGCCTTTTCACAGGCTC -3′  Reverse: 5′- GCTGTTCTCATTGGGTGACTC -3  **TBP**  Forward: 5′- CCATTGCACTTCGTGCCC -3′  Reverse: 5′- GCCAGTCTGGACTGTTCCTC -3 |
